# Fibroblast-specific deletion of IL-1 receptor-1 reduces adverse cardiac remodeling following myocardial infarction

**DOI:** 10.1172/jci.insight.125074

**Published:** 2019-09-05

**Authors:** Sumia A. Bageghni, Karen E. Hemmings, Nadira Y. Yuldasheva, Azhar Maqbool, Filomena O. Gamboa-Esteves, Neil E. Humphreys, Maj Simonsen Jackson, Christopher P. Denton, Sheila Francis, Karen E. Porter, Justin F.X. Ainscough, Emmanuel Pinteaux, Mark J. Drinkhill, Neil A. Turner

**Affiliations:** 1 Discovery and Translational Science Department, Leeds Institute of Cardiovascular and Metabolic Medicine, School of Medicine, University of Leeds, Leeds, United Kingdom.; 2 Leeds Institute of Cancer and Pathology, Leeds Teaching Hospitals NHS Trust, Leeds, United Kingdom.; 3 Faculty of Biology, Medicine and Health, University of Manchester, Manchester, United Kingdom.; 4 Institute of Immunity and Transplantation, Centre for Rheumatology and Connective Tissue Diseases, University College London and Royal Free Hospital, London, United Kingdom.; 5 Department of Infection, Immunity & Cardiovascular Disease, Medical School, University of Sheffield, Sheffield, United Kingdom.

**Keywords:** Cardiology, Inflammation, Cytokines, Fibrosis, Heart failure

## Abstract

It has been hypothesized that IL-1α is released from damaged cardiomyocytes following myocardial infarction (MI) and activates cardiac fibroblasts via its receptor (IL-1R1) to drive the early stages of cardiac remodeling. This study aimed to definitively test this hypothesis using cell type–specific IL-1α and IL-1R1–KO mouse models. A floxed *Il1a* mouse was created and used to generate a cardiomyocyte-specific IL-1α–KO (MIL1AKO) mouse line. A tamoxifen-inducible fibroblast-specific IL-1R1 hemizygous KO (FIL1R1KO) mouse line was also generated. Mice underwent experimental MI (permanent left anterior descending coronary artery ligation), and cardiac function was determined 4 weeks later by conductance pressure-volume catheter analysis. Molecular markers of remodeling were evaluated at various time points by real-time RT-PCR and histology. MIL1AKO mice showed no difference in cardiac function or molecular markers of remodeling after MI compared with littermate controls. In contrast, FIL1R1KO mice showed improved cardiac function and reduced remodeling markers after MI compared with littermate controls. In conclusion, these data highlight a key role for the IL-1R1/cardiac fibroblast signaling axis in regulating remodeling after MI and provide support for the continued development of anti–IL-1 therapies for improving cardiac function after MI. Cardiomyocyte-derived IL-1α was not an important contributor to remodeling after MI in this model.

## Abstract 2

Fibroblast-specific knockout of IL-1R1, but not cardiomyocyte-specific knockout of IL-1α, improved cardiac function and remodeling after myocardial infarction.

## Introduction

Although widely recognized as key regulators of extracellular matrix (ECM) turnover, cardiac fibroblasts also have wide-reaching functions that are fundamental to the overall physiology and pathophysiology of the heart, including sensing tissue damage and contributing to cardiac inflammation (1, 2). Cardiac fibroblasts are particularly responsive to the proinflammatory cytokine IL-1 (3), whose levels are increased with many cardiovascular pathologies, including myocardial infarction (MI), cardiomyopathy, myocarditis, and hypertension (4, 5). In response to IL-1 stimulation, cardiac fibroblasts secrete an array of proinflammatory cytokines and chemokines, and enhance ECM turnover through increased secretion of matrix metalloproteinases (MMPs), decreased collagen synthesis, and decreased expression of profibrotic cytokines (3). Thus, fibroblasts can contribute to the inflammatory milieu that occurs in the myocardium early post-MI, and the IL-1/cardiac fibroblast signaling axis is a potentially important mediator of cardiac remodeling post-MI (2, 3). Furthermore, there is growing evidence to suggest that IL-1 blockade is a viable therapeutic strategy for treating cardiovascular disease (6–8).

IL-1 comprises 2 distinct gene products (IL-1α and IL-1β) that have generally indistinguishable biological activities and play a significant role in the pathogenesis of heart disease (4). Despite their similarity of action, IL-1α and IL-1β are produced by cells through very different mechanisms. IL-1α is an intracellular cytokine (nuclear and cytosolic) that is released when cells undergo necrosis and was originally shown to be a key trigger for inflammation in the liver (9). Importantly, it has also been proposed that IL-1α release from necrotic cardiomyocytes acts in a similar manner, triggering the early inflammatory response post-MI (10). This key role for IL-1α in inflammation post-MI has been supported by a recent murine study in which an IL-1α–specific blocking antibody reduced myocardial injury and improved cardiac function after experimental MI (11). In contrast to IL-1α, IL-1β is inactive until it undergoes inflammasome-mediated proteolytic processing prior to nonclassical secretion from cells in response to specific stimuli (12). In mice, cardiomyocytes express both IL-1α and IL-1β, whereas cardiac fibroblasts express IL-1β but not IL-1α (10).

Irrespective of their differences in expression, synthesis, activation, and secretion, the biological effects of both IL-1α and IL-1β are mediated through the same receptor, IL-1 receptor-1 (IL-1R1). Ligand binding to IL-1R1 stimulates its association with the IL-1 receptor accessory protein (IL-1RacP), which in turn recruits the MyD88 and IRAK4 signaling proteins to form a stable tetrameric signaling module in which all components are essential (13). The downstream functional effects of IL-1 are mediated primarily via the MAP kinase (ERK, p38, JNK) and NF-κB signaling pathways, which are stimulated following recruitment of further adaptor proteins and kinases to the core IL-1R1 signaling module (3, 13).

Studies of IL-1R1 global KO mice have revealed a critical role for IL-1R1 in mediating cardiac myofibroblast accumulation, MMP expression, and fibrotic remodeling of the infarcted heart (14–16). The beneficial effects observed in IL-1R1–KO mice were largely the result of suppressed inflammation; however, direct effects on cardiac fibroblasts were also suggested (14). Nevertheless, the global nature of these IL-1R1–KO models makes it difficult to evaluate the precise contribution of IL-1 signaling in cardiac fibroblasts.

Taken together, these in vivo and in vitro studies suggest that interplay between IL-1α (released from damaged cardiomyocytes) and IL-1R1 activation (on cardiac fibroblasts) is critical in the early myocardial remodeling process post-MI. However, to date no studies to our knowledge have sought to directly evaluate the importance of these interactions in vivo in a cell-specific context. The aim of this study was to use cell type–specific KO mouse models to investigate the role of cardiomyocyte IL-1α and fibroblast IL-1R1 in regulating cardiac remodeling post-MI.

## Results

### Cardiomyocyte-specific IL-1α KO does not affect cardiac function or remodeling after experimental MI.

**Figure 1:**
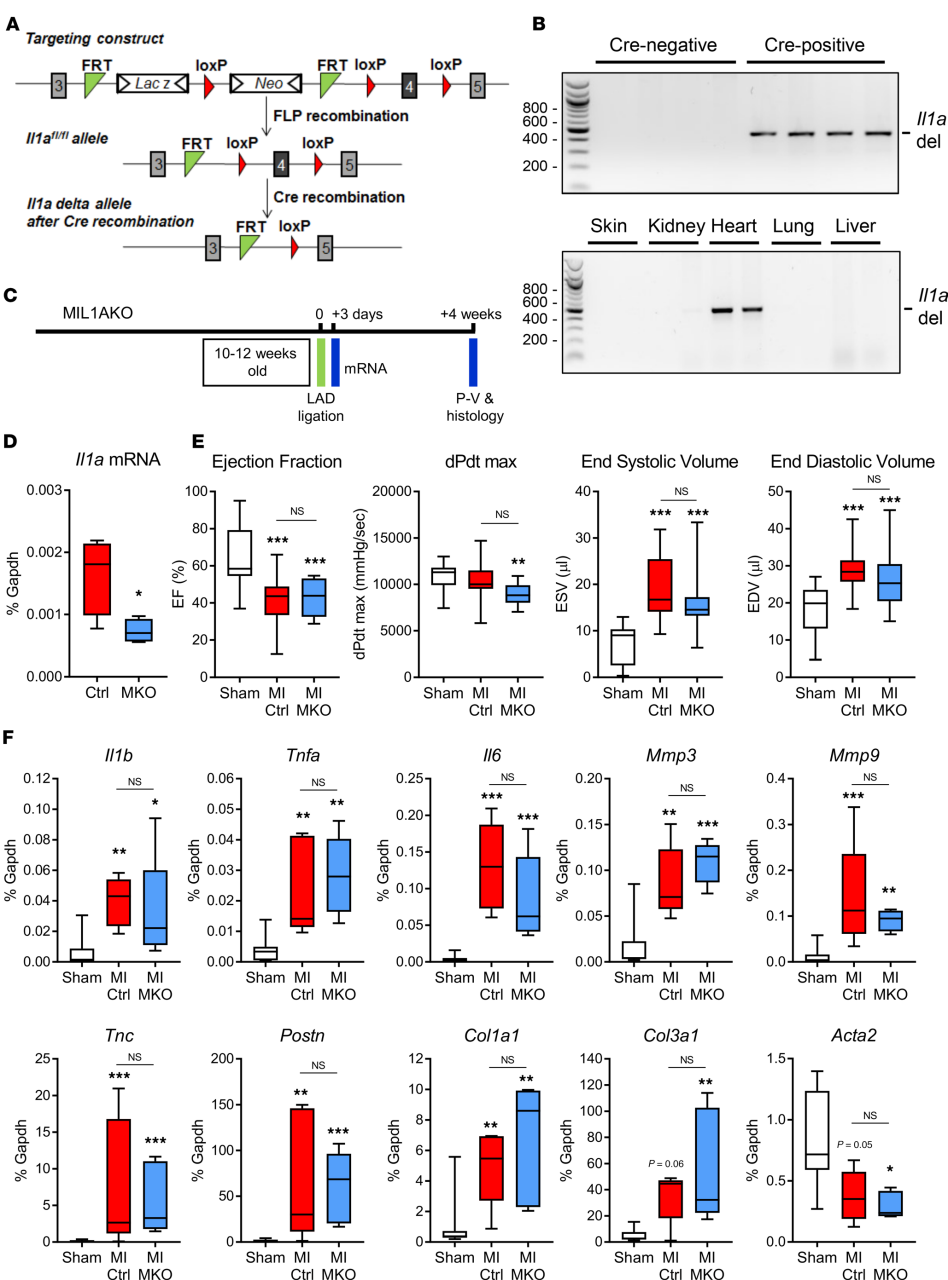
Creation, characterization, and functional assessment of cardiomyocyte-specific IL-1α–KO mouse line (MIL1AKO). (**A**) Generation of floxed IL-1α (exon 4) mice (*Il1a*^fl/fl^). Genetic targeting shows exon 4 flanked by loxP sites, positioned upstream of a lacZ/Neo resistance cassette, which is excised upon Flp recombination, resulting in the generation of *Il1a*^fl/fl^ allele. Cre recombinase leads to exon 4 deletion, generating IL-1 KO mice. (**B**) Genotyping showing exon 4 deletion of *Il1a* gene, with size markers (bp) to the left. Top: PCR analysis for *Il1a* exon 4 deletion (456 bp) in cardiac tissue from Cre-negative *Il1a*^fl/fl^ and *Myh6*-Cre–positive *Il1a*^fl/fl^ (MIL1AKO) mice. Bottom: PCR analysis for exon 4 deletion (456 bp) in various tissues in Cre-positive MIL1AKO mice, confirming cardiac-specific deletion. See Supplemental Table 3 for primer details. (**C**) Timeline showing experimental myocardial infarction (MI) induced by ligation of the left anterior descending (LAD) coronary artery and time points for collection of RNA, histology, and measurement of cardiac function by pressure-volume (P-V) conductance catheter. (**D**) qRT-PCR analysis of basal *Il1a* mRNA expression in hearts from control (Ctrl; Cre-negative *Il1a*^fl/fl^) and MIL1AKO (MKO; *Myh6*-Cre–positive *Il1a*^fl/fl^) mice. *P < 0.05 for effect of KO (unpaired *t* test, *n* = 4). (**E**) P-V conductance catheter data. Sham, mixed genotypes (*n* = 19); MI Ctrl, Cre-negative *Il1a*^fl/fl^ after MI (*n* = 19); MI MKO, *Myh6*-Cre–positive *Il1a*^fl/fl^ (MIL1AKO) after MI (*n* = 17). *P < 0.05; **P < 0.01; ***P < 0.001; NS, not significant between the 2 MI groups (1-way ANOVA with Tukey post hoc). (**F**) qRT-PCR data showing relative mRNA levels of remodeling genes *Il1b*, *Tnfa*, *Il6*, *Mmp3*, *Mmp9*, tenascin C (*Tnc*), α-smooth muscle actin (*Acta2*), periostin (*Postn*), collagen Iα1 (*Col1a1*), and collagen IIIα1 (*Col3a1*). Sham, mixed genotypes (*n* = 7); MI Ctrl, Cre-negative *Il1a*^fl/fl^ after MI (*n* = 5); MI MKO, *Myh6*-Cre–positive *Il1a*^fl/fl^ (MIL1AKO) after MI (*n* = 5). *P < 0.05; **P < 0.01; ***P < 0.001 versus sham; NS, not significant between MI groups (1-way ANOVA with Tukey post hoc).

A cardiomyocyte-specific IL-1α–KO mouse model was generated to evaluate the contribution of cardiomyocyte-derived IL-1α to cardiac remodeling post-MI. Firstly, a floxed *Il1a* mouse was created in which exon 4 of the *Il1a* gene was modified to be flanked by loxP sites (Figure 1A and Supplemental Figure 1A; supplemental material available online with this article; https://doi.org/10.1172/jci.insight.125074DS1). Floxed *Il1a* mice were then bred with mice expressing Cre recombinase under control of the cardiomyocyte-specific *Myh6* promoter to produce Cre-positive cardiomyocyte-specific IL-1α–KO (MIL1AKO) mice and control Cre-negative floxed littermates (Supplemental Figure 1B). Deletion PCR confirmed that Cre-positive MIL1AKO mice had the expected deletion within exon 4 of the *Il1a* gene and that this was only apparent in heart tissue (Figure 1B). Mean *Il1a* mRNA expression levels in whole heart samples from control mice were relatively low and were further reduced in hearts from MIL1AKO mice (Figure 1D), reflecting *Il1a* gene deletion in the cardiomyocyte population of the heart. Baseline cardiac characteristics in control (Cre-negative *Il1a*^fl/fl^) and MIL1AKO (Cre-positive *Il1a*^fl/fl^) mice were assessed by pressure-volume (P-V) conductance catheter analysis and showed no difference between groups (Supplemental Figure 2A). Basal expression levels of remodeling genes (collagens and hypertrophy markers) were also similar for the 2 lines (Supplemental Figure 2B). Thus, Cre-induced deletion of IL-1α in cardiomyocytes had no effect on the baseline cardiac phenotype of the animals.

**Figure 2:**
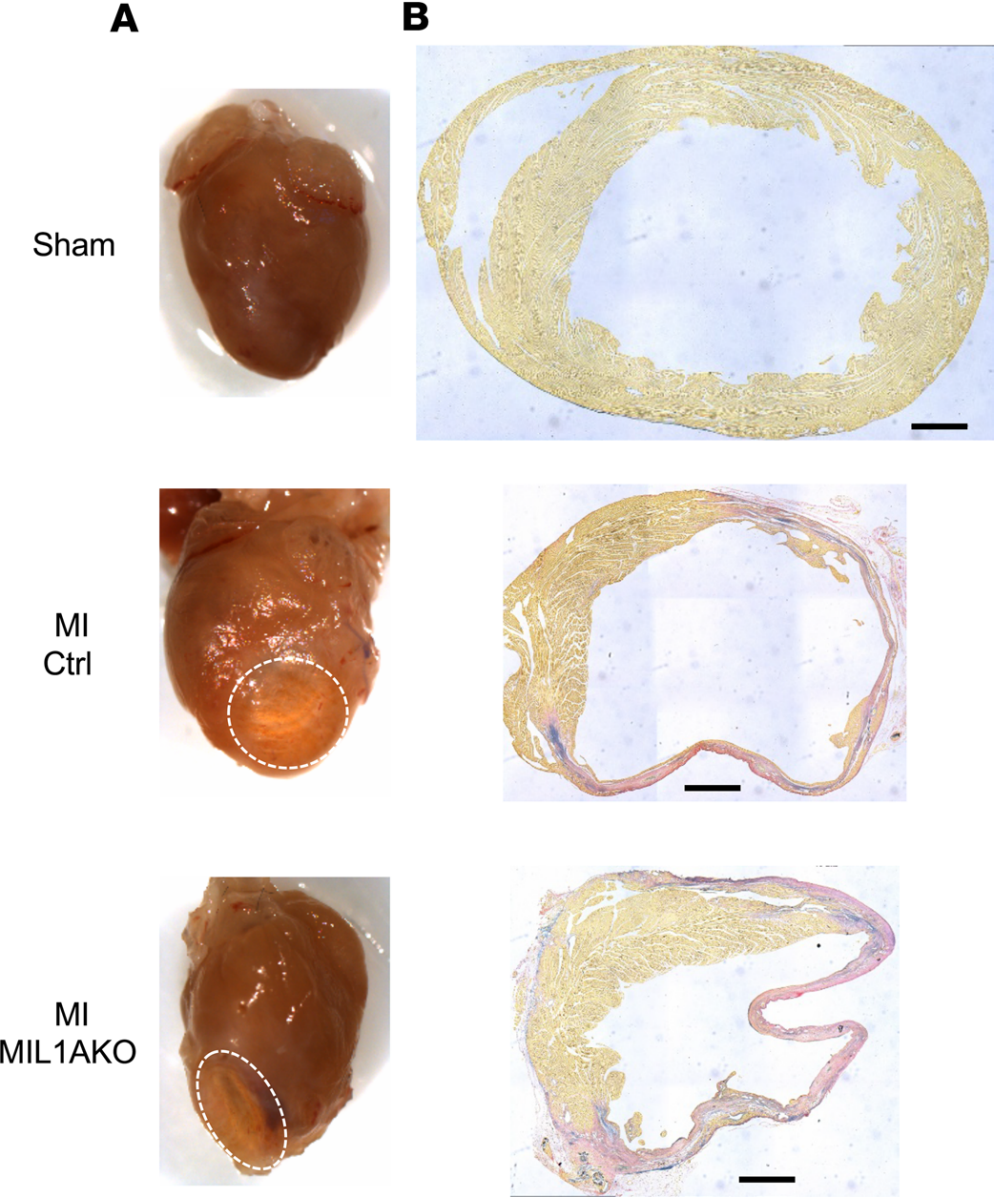
Effect of cardiomyocyte-specific IL-1α deletion on cardiac remodeling after MI. (**A**) Representative images of sham and myocardial infarction (MI) hearts at the end of the experimental period (4 weeks after surgery). Sham, mixed genotypes; MI Ctrl, Cre-negative *Il1a*^fl/fl^ after MI; MI MIL1AKO, *Myh6*-Cre–positive *Il1a*^fl/fl^ after MI (*n* = 5). Dashed line, infarcted area. (**B**) Representative photomicrographs of histological sections of hearts stained with Miller’s elastic van Gieson. Yellow stain, muscle; pink, collagen; purple, elastin. Scale bars: 700 μm.

To determine the effect of cardiomyocyte-specific IL-1α KO on post-MI function and remodeling, we used a permanent left anterior descending (LAD) coronary artery ligation model of experimental MI. Male Cre-negative (control) and Cre-positive (MIL1AKO) littermates underwent LAD ligation at 10–12 weeks of age (Figure 1C), and cardiac physiological measurements were obtained 4 weeks later by P-V conductance catheter analysis (Figure 1E). MI scarring was clearly visible in hearts obtained from animals that had undergone LAD ligation (Figure 2A).

As expected, control mice displayed overt cardiac dysfunction 4 weeks post-MI, as evidenced by reduced ejection fraction (EF), increased end-systolic volume (ESV), and increased end-diastolic volume (EDV) compared with sham animals (Figure 1E), with other parameters being unaffected (Supplemental Table 1). Importantly, no difference in post-MI cardiac function was observed between control mice and MIL1AKO mice (Figure 1E and Supplemental Table 1), indicating that cardiomyocyte-derived IL-1α per se was not an important contributor to cardiac dysfunction post-MI.

Assessment of gene expression in the infarct region 3 days post-MI by real-time quantitative reverse-transcription PCR (qRT-PCR) revealed increases in mRNA levels of inflammatory genes (*Il1b*, *Tnfa*, *Il6*) and ECM regulatory genes (*Mmp3*, *Mmp9, Tnc, Postn, Col1a1, Col3a1*) and reduced mRNA for the myofibroblast differentiation marker *Acta2* in MI hearts compared with sham hearts (Figure 1F). Importantly, there was no difference in expression levels of these genes between control and MIL1AKO mice post-MI (Figure 1F). Histological analysis of hearts 4 weeks post-MI revealed no obvious differences between control and MIL1AKO mice, with both showing thinning of the LV free wall in the infarct area and scar tissue marked by ECM deposition (Figure 2B), in agreement with the functional and mRNA expression data.

### Characterization of fibroblast-specific IL-1R1–KO mouse model.

Having ruled out a role for cardiomyocyte-derived IL-1α in modulating cardiac remodeling post-MI, we proceeded to investigate the specific role of the IL-1 receptor (IL-1R1) expressed by cardiac fibroblasts. *Col1a2*-CreER(T) mice have previously been used to induce Cre-lox-mediated deletion of genes specifically in fibroblasts (17), including those in the heart (18–20), without affecting cardiomyocytes, endothelial cells, smooth muscle cells, progenitor cells, pericytes, or macrophages. We first developed a *Col1a2*-Cre-*Il1r1*^fl/fl^ mouse line (see Supplemental Figure 3A for breeding strategy) to explore the effects of fibroblast-specific IL1R1 deletion on cardiac remodeling. However, this method was ineffective at reducing IL1R1 expression or activation in cardiac fibroblasts (data not shown), despite our previous success with this approach for deleting other genes (20). To overcome this problem, we adopted a modified strategy to generate a hemizygous mouse line with global deletion of one *Il1r1* allele in which the remaining allele was floxed. This approach is particularly useful for targeting genes whose heterozygous (+/–) KO does not cause a phenotype, as it maximizes the effect of inducible KO of the remaining allele (21).

**Figure 3:**
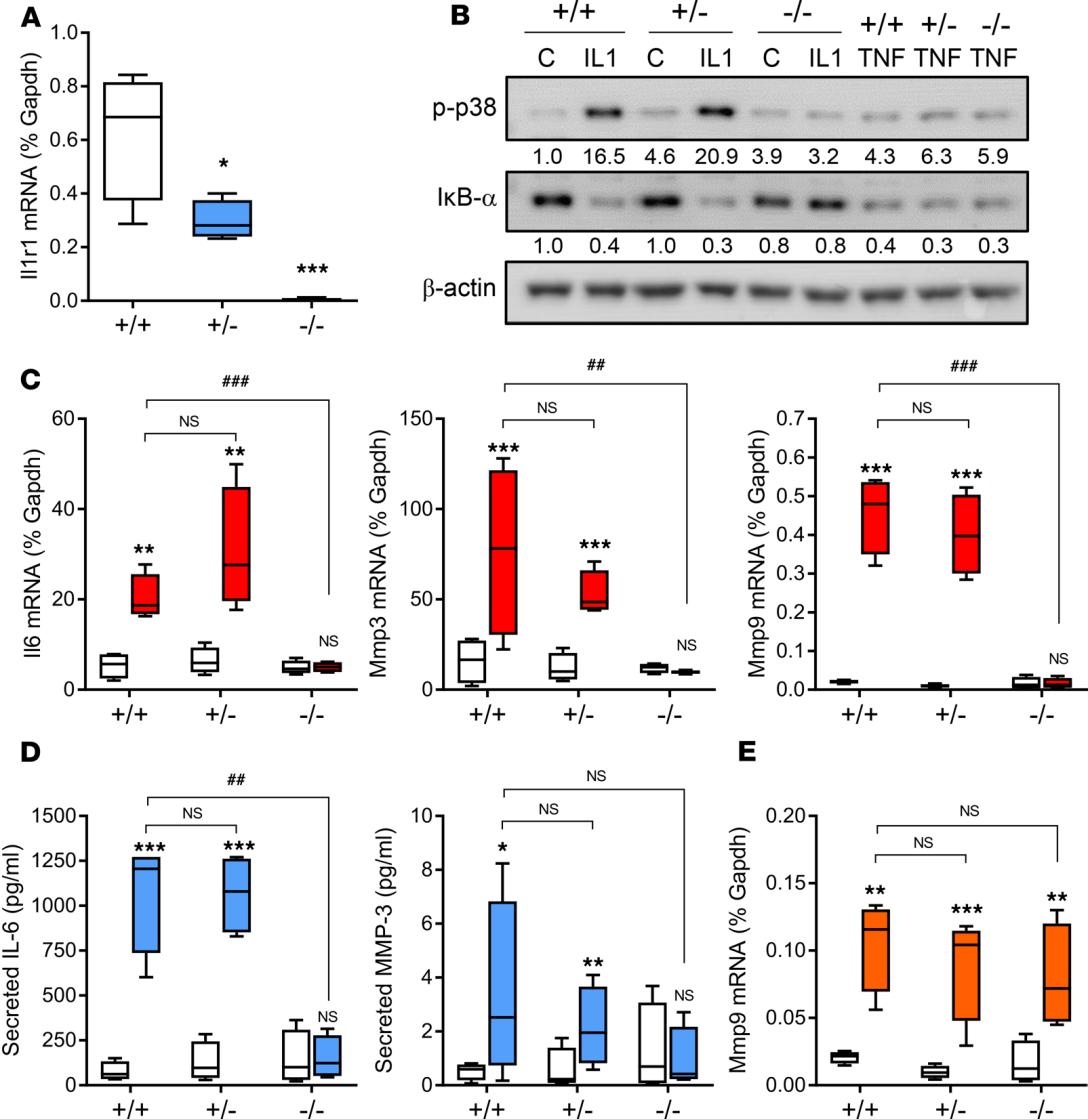
Effect of global IL-1R1 KO on cardiac fibroblast responses to inflammatory cytokines. (**A**) qRT-PCR data showing basal IL-1 receptor 1 (*Il1r1*) mRNA levels in cardiac fibroblasts cultured from wild-type (+/+), IL-1R1 heterozygote (+/–), and IL-1R1–KO (–/–) mice (all *n* = 4). *P < 0.05; ***P < 0.001 versus wild-type (1-way ANOVA with Tukey post hoc). (**B**) Cardiac fibroblasts were exposed to vehicle control (C), 1 ng/ml IL-1α (IL1), or 10 ng/ml TNF-α (TNF) for 30 minutes. Immunoblotting was used to measure activation of p38 MAP kinase (phosphorylation; p-p38) or activation of IκB-α (proteasome-mediated degradation). β-Actin, loading control. Numbers under blots represent densitometric analysis relative to β-actin normalized to lane 1. Representative of 2 separate experiments. (**C** and **D**) Cardiac fibroblasts were treated with vehicle (white boxes) or 1 ng/ml IL-1α (colored boxes) for 6 hours before measuring mRNA levels of *Il6*, *Mmp3*, and *Mmp9* by qRT-PCR (**C**) and protein secretion by ELISA (**D**). (**E**) Cardiac fibroblasts were treated with vehicle (white boxes) or 10 ng/ml TNF-α (colored boxes) for 6 hours before measuring *Mmp9* mRNA levels by qRT-PCR. *P < 0.05; **P < 0.01; ***P < 0.001, NS, not significant for effect of cytokine. ^###^*P* < 0.001; ^##^*P* < 0.01, NS, not significant for effect of IL-1R1 KO (2-way ANOVA with Bonferroni post hoc). *n* = 4 per genotype.

Therefore, we first generated global IL-1R1^+/–^ heterozygous mice and IL-1R1^–/–^ KO mice (see Supplemental Figure 3B for breeding strategy) to investigate the phenotypic effect of monoallelic and biallelic deletion of *Il1r1*. As expected, primary cultures of cardiac fibroblasts from heterozygous animals exhibited a 50% reduction in *Il1r1* mRNA levels compared with wild-type mice, with no detectable expression in those from IL-1R1–KO mice (Figure 3A). The functional effect of fibroblast IL1R1 KO was evaluated in cultured cardiac fibroblasts by measuring IL-1α responses, specifically kinase signaling pathways, gene expression, and protein secretion. IL-1α stimulated the p38 and NF-κB signaling pathways (Figure 3B) and increased *Il6*, *Mmp3*, and *Mmp9* mRNA expression (Figure 3C) and IL-6 and MMP-3 protein secretion (Figure 3D) to a similar extent in fibroblasts from wild-type and heterozygous mice, despite the latter expressing only half the amount of IL-1R1. Cardiac fibroblasts from IL-1R1–KO mice did not respond to IL-1α stimulation (Figure 3, B–D), confirming full loss of the receptor. Responses to TNF-α, a proinflammatory cytokine that activates similar signaling pathways and gene expression profiles to IL-1α but via a different receptor, were unaffected in cardiac fibroblasts from IL-1R1–KO mice (Figure 3, B and E). Together, these data establish that full biallelic deletion of *Il1r1* is necessary to disrupt IL-1 responses in cardiac fibroblasts and that monoallelic deletion is ineffective.

**Figure 4:**
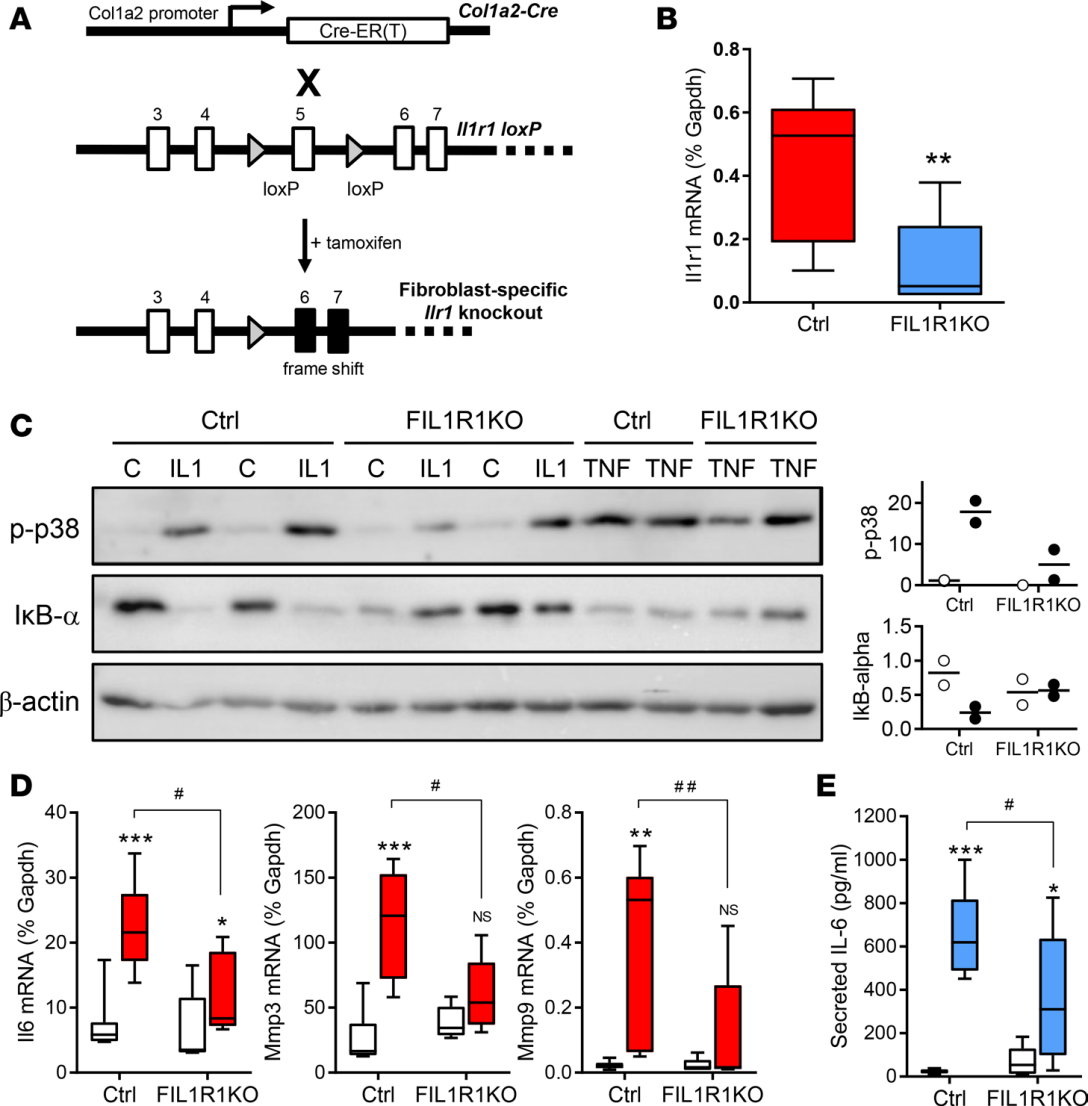
Effect of tamoxifen-induced fibroblast-specific IL-1R1 knockdown on cardiac fibroblast responses to IL-1. (**A**) Schematic showing loxP-targeted Cre-mediated deletion of exon 5 within the IL-1 receptor-1 gene, *Il1r1*. (**B**–**E**) Fibroblast cultures were established from hearts of Cre-negative (Ctrl; *n* = 7) and Cre-positive (fibroblast-specific IL-1 receptor 1 KO [FIL1R1KO]; *n* = 5) hemizygous *Il1r1*^fl/–^ mice 6 weeks after tamoxifen injection. (**B**) qRT-PCR analysis of basal *Il1r1* mRNA expression in cardiac fibroblasts from Ctrl and FIL1R1KO mice. **P < 0.01 for effect of KO (unpaired *t* test). (**C**) Cardiac fibroblasts with different genotypes were exposed to vehicle (control, **C**), IL-1α (IL-1), or TNF-α (TNF) for 30 minutes before immunoblotting to measure activation of p38 MAP kinase (phosphorylation; p-p38) or activation of IκB-α (proteasome-mediated degradation). β-Actin, loading control. Plots to the right depict densitometric analysis relative to β-actin normalized to lane 1 for control (white symbols) or IL-1 treatment (colored symbols). *n* = 2. (**D** and **E**) Cardiac fibroblasts were treated with vehicle (white boxes) or 1 ng/ml IL-1α (colored boxes) for 6 hours before measuring mRNA levels of *Il6*, *Mmp3*, and *Mmp9* by qRT-PCR (**D**), and IL-6 secretion by ELISA (**E**). ***P < 0.001; **P < 0.01; *P < 0.05, NS, not significant for effect of IL-1; ^##^*P* < 0.01; ^#^*P* < 0.05 for effect of IL-1R1 KO (2-way ANOVA with Bonferroni post hoc).

We next generated hemizygous mice in which one *Il1r1* allele was floxed and the other was deleted (fl/–). These animals were crossed with heterozygous mice that expressed tamoxifen-inducible Cre recombinase [CreER(T)] under the control of the fibroblast-specific *Col1a2* promoter (Figure 4A) to generate *Col1a2*-CreER(T)-*Il1r1*^fl/–^ mice, a line we named FIL1R1KO (see Supplemental Figure 3C for breeding strategy). Example genotyping results are shown in Supplemental Figure 4. Mice were injected with tamoxifen at 12 days of age to induce Cre-loxP–-mediated deletion, and hearts isolated after a further 4–8 weeks for analysis. The efficiency of fibroblast-specific deletion of IL-1R1 was confirmed in primary cultures of cardiac fibroblasts by qRT-PCR. Cells from FIL1R1KO mice had a mean 75% reduction in basal *Il1r1* mRNA levels compared with cells from Cre-negative *Il1r1*^fl/–^ littermate control mice (Figure 4B), indicating that full biallelic deletion of *Il1r1* had occurred in response to tamoxifen in approximately 75% of cardiac fibroblasts. The functional effect of IL-1R1 deletion was evidenced by a marked reduction in the activation of the p38 and NF-κB signaling pathways in response to IL-1α, without affecting TNF-α–induced signaling (Figure 4C). Moreover, mean IL-1α–induced *Il6*, *Mmp3*, and *Mmp9* mRNA expression was reduced by 65%–75% in fibroblasts cultured from FIL1R1KO hearts compared with cells from control littermate hearts (Figure 4D), in close agreement with the extent of IL1R1 knockdown (Figure 4B). Similar changes were noted for protein secretion; for example, mean IL-1α–induced IL-6 secretion was reduced by > 50% in cardiac fibroblasts from FIL1R1KO mice (Figure 4E).

### Fibroblast-specific IL-1R1 KO improves cardiac remodeling after experimental MI.

**Figure 5:**
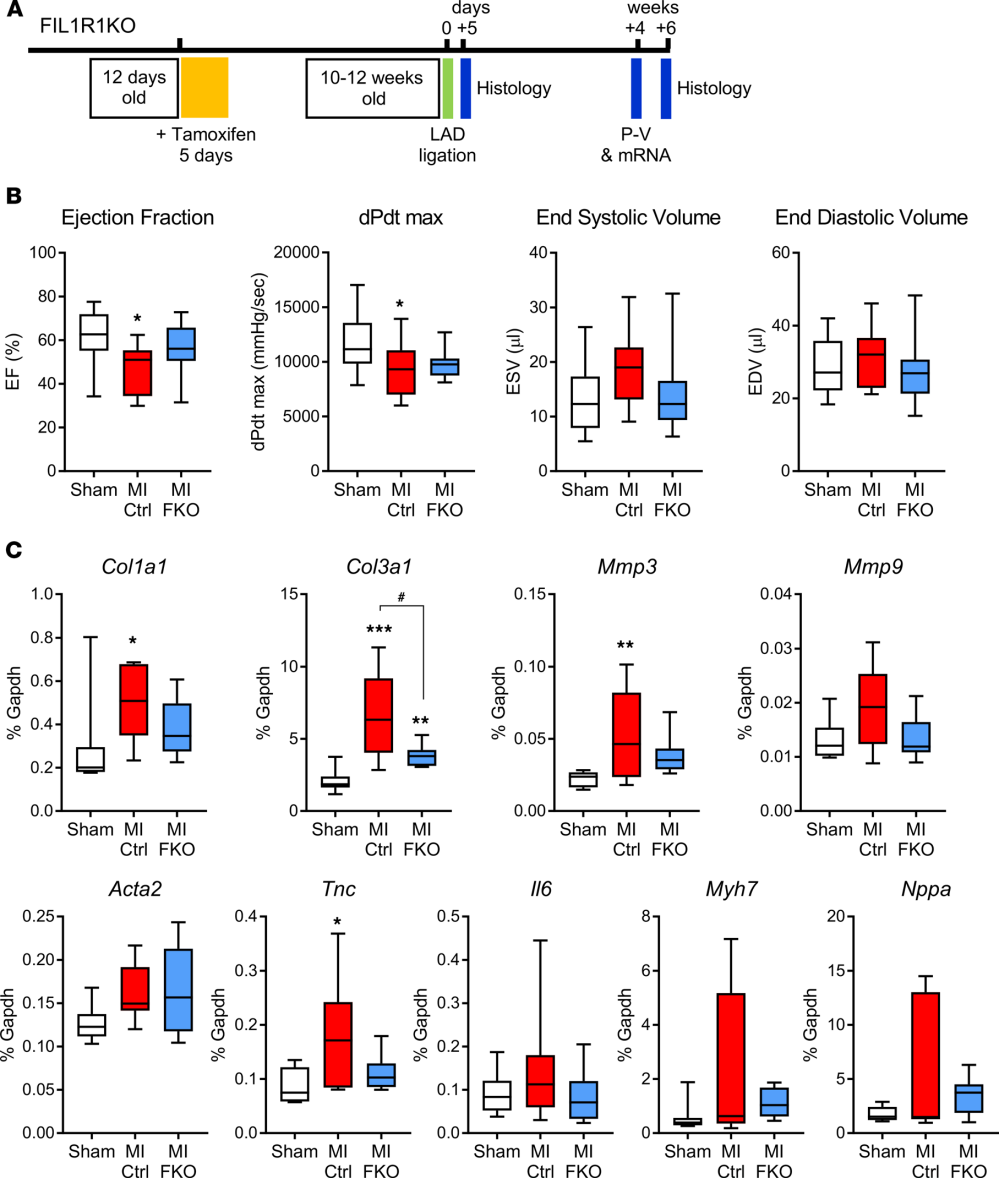
Effect of fibroblast-specific IL-1R1 deletion on cardiac function and remodeling genes 4 weeks after MI. (**A**) Experimental timeline showing timing of tamoxifen injection and experimental myocardial infarction (MI) induced by ligation of the left anterior descending (LAD) coronary artery, as well as the timing for histology, collection of RNA, and measurement of cardiac function by pressure-volume (P-V) conductance catheter. (**B**) P-V conductance catheter data. Sham, mixed genotypes, no tamoxifen treatment (*n* = 18); MI Ctrl, tamoxifen-treated Cre-negative *Il1r1*^fl/–^ after MI (*n* = 11); MI FKO, tamoxifen-treated Cre-positive *Il1r1*^fl/–^ (FIL1R1KO; fibroblast-specific IL-1 receptor 1 KO) after MI (*n* = 11). *P < 0.05 versus sham. (**C**) qRT-PCR data showing relative mRNA levels of remodeling genes collagen Iα1 (*Col1a1*), collagen IIIα1 (*Col3a1*), *Mmp3*, *Mmp9*, α-smooth muscle actin (*Acta2*), tenascin C (*Tnc*), *Il6*, β-myosin heavy chain (*Myh7*), and atrial natriuretic factor (*Nppa*). Sham, mixed genotypes, no tamoxifen treatment (*n* = 8); MI Ctrl, tamoxifen-treated Cre-negative mice after MI (*n* = 8); MI FKO, tamoxifen-treated FIL1R1KO mice after MI (*n* = 8). *P < 0.05; **P < 0.01; ***P < 0.001 versus sham; ^#^*P* < 0.05 versus MI Ctrl (1-way ANOVA with Tukey post hoc).

Having confirmed functional and sustained knockdown of IL-1R1 in cardiac fibroblasts isolated from tamoxifen-treated FIL1R1KO mice, we next investigated the in vivo effect of fibroblast-selective IL-1R1 deletion on cardiac function after experimental MI (Figure 5A). Male Cre-negative (control) and Cre-positive (FIL1R1KO) littermates were injected with tamoxifen at 12 days of age for 5 consecutive days and underwent permanent LAD coronary artery ligation surgery at 10–12 weeks of age. Sham control animals (pooled Cre-negative and Cre-positive) were not injected with tamoxifen. There were no differences in baseline cardiac characteristics between Cre-positive and Cre-negative mice (Supplemental Figure 5). Physiological measurements were obtained 4 weeks after LAD ligation by P-V catheter analysis, before hearts were analyzed for histological and molecular markers of remodeling. P-V analysis (Figure 5B and Supplemental Table 2) revealed significantly reduced EF in control mice 4 weeks post-MI compared with sham (48% versus 61% respectively; *P* = 0.02), but, importantly, EF was only partially reduced to 56% in FIL1R1KO mice post-MI and was not significantly different than that of sham controls. dPdtmax (an indicator of contractility) was also significantly decreased in control MI mice compared with sham animals but was less affected in FIL1R1KO mice; the difference in dPdtmax between post-MI FIL1R1KO and sham animals was not significant. Similar, nonsignificant trends were observed with ESV and EDV (Figure 5B and Supplemental Table 2). End-systolic pressures were significantly reduced in MI mice of both genotypes compared with sham animals, whereas other cardiac parameters and arterial blood pressure were not significantly different between the 3 groups (Supplemental Table 2).

The impact of fibroblast-selective IL-1R1 deletion on specific molecular markers of cardiac remodeling was assessed by qRT-PCR of whole ventricle mRNA samples taken from hearts used for P-V analysis (i.e., 4 weeks after LAD ligation). Nine genes were studied that included markers of ECM synthesis (*Col1a1*, *Col3a1*), ECM degradation (*Mmp3*, *Mmp9*), myofibroblast activation (*Acta2*), mechanical activation (*Tnc, Il6*), and cardiomyocyte hypertrophy (*Myh7*, *Nppa*). Significant increases in *Col1a1* (1.7-fold increase; *P* < 0.05), *Col3a1* (3.2-fold; *P* < 0.001), *Mmp3* (2.5-fold; *P* < 0.01), and *Tnc* (2.1-fold; *P* < 0.01) mRNA levels were observed in control hearts after MI compared with sham hearts (Figure 5C). By comparison, the expression levels of 4 of these genes was markedly less in hearts after MI from FIL1R1KO mice [*Col1a1* (1.3-fold increase), *Col3a1* (1.8-fold; *P* < 0.05 versus control MI), *Mmp3* (1.7-fold), *Tnc* (1.3-fold)] and, with the exception of *Col3a1*, were not significantly different from sham levels (Figure 5C).

**Figure 6:**
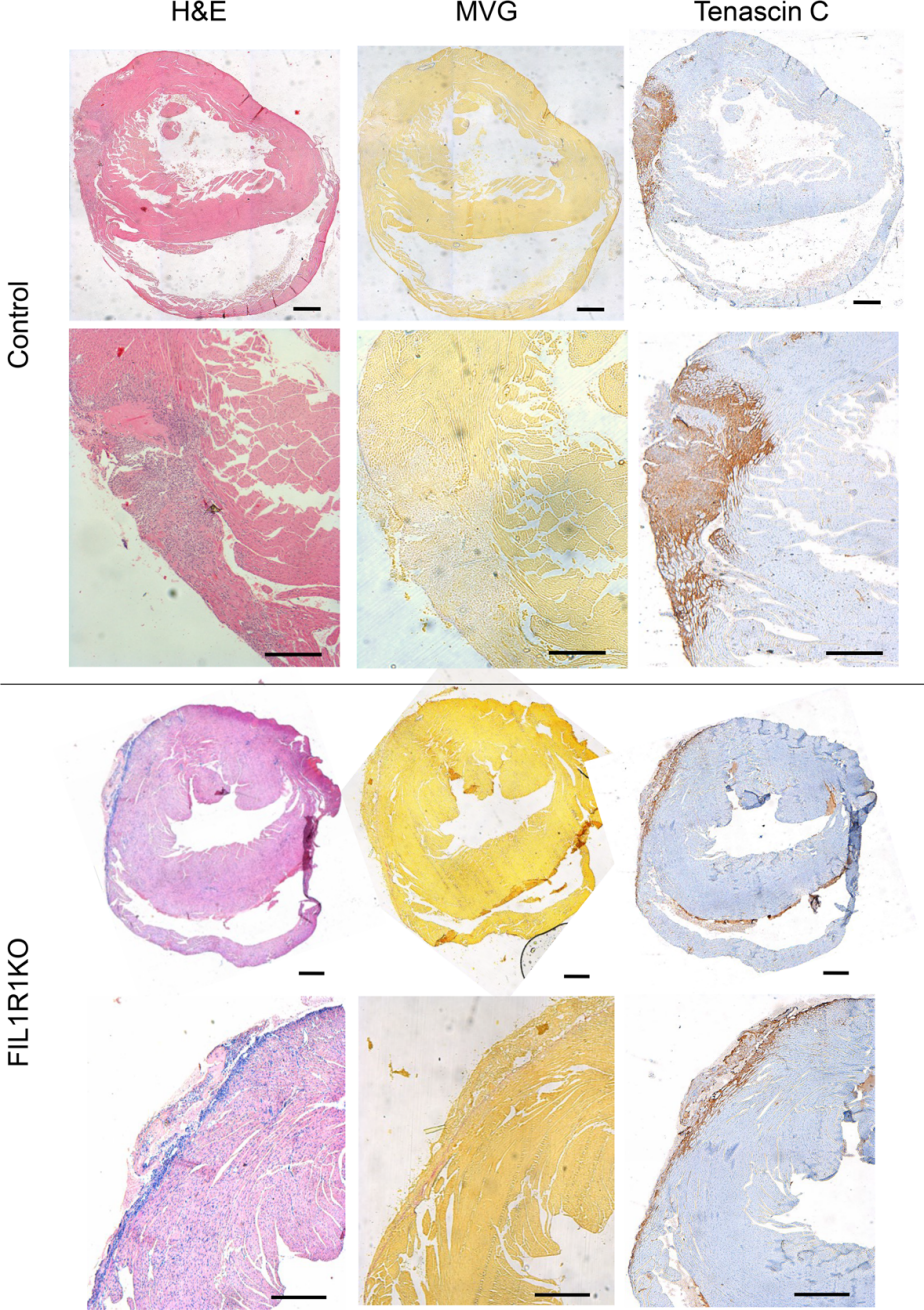
Effect of fibroblast-specific IL-R1 deletion on histological markers of cardiac remodeling 5 days after MI. Representative photomicrographs of adjacent histological sections from control and fibroblast-specific IL-1 receptor 1–KO (F1L1RKO) hearts. Left column: H&E staining showing inflammatory infiltrates and interstitial cells in the infarct zone. Middle column: Miller’s elastic van Gieson (MVG) staining showing elastin (blue) and collagen (pink) staining; muscle tissue is stained yellow. Right column: Tenascin C staining (brown). Scale bars: 500 μm.

**Figure 7:**
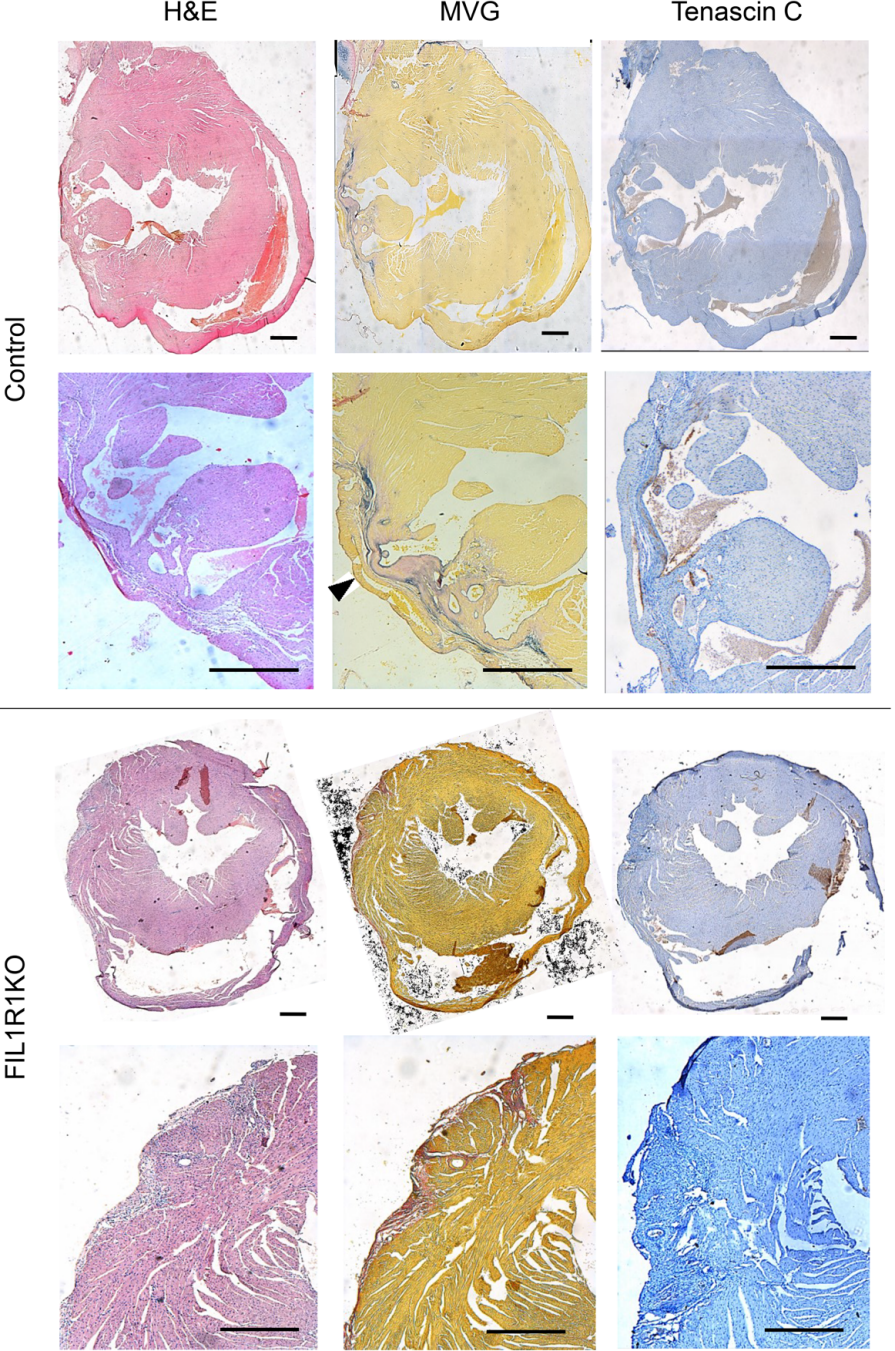
Effect of fibroblast-specific IL-R1 deletion on histological markers of cardiac remodeling 6 weeks after MI. Representative photomicrographs of adjacent histological sections from control and fibroblast-specific IL-1 receptor 1–KO (F1L1RKO) hearts. Left column: H&E staining showing inflammatory infiltrates and interstitial cells in the infarct zone. Middle column: Miller’s elastic van Gieson (MVG) staining showing elastin (blue) and collagen (pink) staining; muscle tissue is stained yellow. The area of ventricular thinning is indicated with an arrowhead. Right column: Tenascin C staining (brown). Scale bars: 500 μm.

Histological staining was performed on hearts 5 days (Figure 6) or 6 weeks (Figure 7) after MI. In control mice, granulation tissue was evidenced after 5 days by inflammatory cell infiltration (Figure 6, H&E) and marked tenascin C expression was also observed (Figure 6), with a small amount of collagen expression (Figure 6, MVG). After 6 weeks, control mice had evidence of ventricular wall thinning (Figure 7) and more collagen deposition (Figure 7, MVG), with very little tenascin C expression (Figure 7). All these changes were more pronounced in control mice compared with FIL1R1KO mice (Figures 6 and 7).

## Discussion

We used two potentially novel cell-specific KO mouse models to investigate the role of cardiomyocyte-derived IL-1α and the cardiac fibroblast IL-1 receptor, IL-1R1, in remodeling of the heart after MI. The principal conclusion of our study was that fibroblast-specific deletion of IL-1R1 was cardioprotective following MI, whereas cardiomyocyte-specific deletion of IL-1α had no effect. Our data with FIL1R1KO mice suggest that fibroblast IL-1R1 signaling is a significant driver of detrimental cardiac remodeling after MI. In vitro studies have revealed that cardiac fibroblasts adopt a proinflammatory, proangiogenic, promigratory, ECM-degrading, nondifferentiated phenotype in response to IL-1 (3). Our in vivo observations suggest that these acute effects of IL-1 on cardiac fibroblasts early after MI contribute to longer-term adverse cardiac remodeling that is detrimental to cardiac function. These data support ongoing strategies aimed at inhibiting IL-1 or its receptor to improve cardiac function after MI (6–8).

### Role of IL-1α.

Cardiomyocyte-specific overexpression of human IL-1 induces cardiac hypertrophy in mice (22). Moreover, it has been proposed by Lugrin and colleagues that cardiomyocyte-derived IL-1α is important for stimulating the early inflammatory response after MI (10). On the contrary, we observed no difference in cardiac function or expression of remodeling genes after MI in a cardiomyocyte-specific IL-1α–KO mouse (MIL1AKO) compared with control mice, suggesting that cardiomyocyte-derived IL-1α is not an essential player in this process. It is worth noting several differences between our study and that of Lugrin et al. (10). First, we used a cardiomyocyte-specific IL-1α–KO mouse, whereas their study used global IL-1α–KO mice, so it is possible that the effects that were observed therein were due to IL-1α deletion in nonmyocyte cells (e.g., endothelial cells, fibroblasts, inflammatory cells), which together account for 70% of cells in the murine heart (23). Second, the experimental MI model used in Lugrin’s study (10) was an ischemia/reperfusion model of MI, as opposed to the permanent LAD ligation model we used. Although the ischemia/reperfusion model more closely resembles the clinical scenario where most patients undergo revascularization, the permanent occlusion model is particularly useful for understanding direct mechanisms without the confounding element of reperfusion. We chose the permanent occlusion model based on previous studies showing that interference of IL-1 signaling had favorable effects in both nonreperfusion (16) and reperfusion models (10, 14). Finally, Lugrin et al. (10) did not evaluate longer-term functional endpoints, with all measurements being taken immediately after the reperfusion period (2 hours). The increase in IL-6, one of the markers studied, was reduced in global IL-1α–KO mice after the 2-hour reperfusion period (10). In contrast, in our study, *Il6* mRNA levels remained significantly elevated 3 days after permanent LAD occlusion and were not affected by cardiomyocyte-specific IL-1 deletion, indicating that there was no long-term effect on inflammation.

Our results do not preclude an important role for IL-1α per se, and, indeed, there is recent evidence that cardiac IL-1α is important in cardiac remodeling after MI, as administration of an IL-1α–neutralizing antibody reduced infarct size and preserved LV systolic function in an ischemia/reperfusion model of MI (11). Although we observed that basal cardiac *Il1a* mRNA levels were very low, and were further reduced in MIL1AKO mice, IL-1α release could still occur in our model in nonmyocyte cells, including endothelial cells, fibroblasts, and macrophages, all of which may contribute to cardiac IL-1α levels after MI.

### Role of IL-1β.

Although our study did not directly investigate the contribution of IL-1β to remodeling after MI, our data with the FIL1R1KO mouse may indicate a role for IL-1β acting on fibroblast IL-1R1. Indeed, such a role is supported by mouse and rat studies in which anti–IL-1β–neutralizing antibodies administered after MI improve cardiac function and limit adverse remodeling (24, 25). IL-1β is produced by multiple cardiac cell types (26) and can directly promote adverse cardiac remodeling (4).

### Clinical relevance.

Therapeutic IL-1 blockade has now been evaluated in several cardiovascular clinical trials (reviewed in refs. 6–8). Initially, a number of small-scale trials investigated the effect of anakinra, a recombinant form of the IL-1 receptor antagonist, on inflammation and cardiac remodeling in stable-STEMI and non-STEMI patients (27, 28) and reported that IL-1 inhibition reduced inflammation after MI but had little effect on cardiac function in the longer term. This may relate to the relatively mild inflammatory phenotype in these patient cohorts compared with general STEMI patients, in whom IL-1 blockade was more effective (29). A more recent anakinra trial (D-HART2) reported that IL-1 blockade did not improve cardiorespiratory fitness in obese HF patients with preserved EF, although markers of systemic inflammation and myocardial strain were reduced (30). In comparison to general IL-1 blockade with anakinra, the recent CANTOS trial used an IL-1β–specific neutralizing antibody (canakinumab) and reported a reduction in cardiovascular events independent of lipid levels (31), with a suggestion that the cardioprotective effects extended beyond new atherothrombotic events. Thus, there is gathering momentum for the use of targeted anti–IL-1 therapies for improving outcome after MI, at least in some patient populations.

### Study limitations.

Although our study provided evidence for a role of fibroblast IL-1R1 in driving adverse cardiac remodeling, it remains unclear whether IL-1α or IL-1β (or both) is the most important stimulus. Our results with MIL1AKO mice ruled out a role for cardiomyocyte-derived IL-1α, but other cellular sources of IL-1α were not explored. Future studies investigating selective IL-1α KO in other cardiac cell types (fibroblasts, endothelial cells, macrophages) will be required to explore this further. Similarly, global or cell-specific IL-1β KO mice would be helpful in determining the precise role of IL-1β in this setting. We used a permanent LAD ligation model of MI; investigations with an ischemia/reperfusion model of MI would be important to confirm our data in a more clinically appropriate model. We did not evaluate the area at risk in our MI models, although this might have been useful to understand the underlying mechanisms for cardioprotection in FIL1R1KO mice. Although we studied specific features of remodeling by quantifying expression levels of specific genes, and provided representative histological images, a more in-depth quantitative analysis of inflammation and fibrosis using supportive methodologies (e.g., flow cytometry and hydroxyproline incorporation, respectively) would have strengthened our conclusions. Prolonged cardiomyocyte-specific Cre expression has been reported to result in modest cardiac dysfunction, fibrosis, and hypertrophy in 6-month-old *Myh6*-Cre mice (32) but not in 3-month-old mice (32, 33). The MIL1AKO mice used in the current study were 10–12 weeks of age at the time of surgery, and, although not directly addressed in our study, it is therefore unlikely that age-dependent Cre cardiotoxicity influenced the data.

### Conclusion.

Our data highlight the importance of the IL-1/cardiac fibroblast signaling axis in regulating remodeling after MI and provide support for the continued development of anti–IL-1 therapies for improving cardiac function after MI.

## Methods

### Animal welfare

Mice were maintained in individually ventilated cages at 21°C, 50%–70% humidity, and 12/12-hour-light/dark cycle, with Pure-o’Cel paper bedding (Datesand) and ad libitum access to water and RM1 diet (Special Diets Services).

### Mouse models

#### Floxed mice.

Floxed *Il1a* (exon 4) mice were created as follows. *Il1a*^tm1a(EUCOMM)Wtsi^ embryonic stem cells (34) were purchased from the European Mouse Mutant Cell Repository. Cells from clone EPD0822-4-H02 were prepared for microinjection according to a previously published protocol (35) with minor modifications. Briefly, cells were cultured in KO-DMEM plus KOSR medium plus 2i (MEK inhibitor and GSK3 inhibitor) on a gelatin-coated (0.1% gelatin in phosphate-buffered saline) cell culture dish maintained under standard culture conditions (37°C, 5% CO_2_, humidified). Culture medium was changed daily, and cells were passaged when 75%–80% confluent using accutase (Sigma-Aldrich) to dissociate them. Cells were passaged no more than 3 times and were transferred to media without 2i reagents for 24 hours before microinjection. Cells were then microinjected into 4–8 cell B6N-Tyr^c–Brd^/BrdCrCrl embryos. Surviving embryos were surgically implanted into the oviduct of day 0.5 postcoitum pseudopregnant mice. Resulting black/white C57BL/6N chimeras were backcrossed onto C57BL/6N wild-type mice to assess germline penetrance. Potential founder mice were screened by PCR for LacZ, Neo, and LoxP sites. This line was further crossed with C57BL/6N-Tg(CAG-Flpo)1Afst/Mmucd mice from the Mutant Mouse Resource & Research Centers. The FLP recombinase expression provided by this line resulted in a “conditional ready” (i.e., floxed) *Il1a*^tm1c(EUCOMM)Wtsi^ allele, in which exon 4 is flanked by loxP sites. Floxed *Il1r1* (exon 5) mice have been described previously (36) and were provided in-house.

#### Cre-expressing mice.

Cardiomyocyte-specific *Myh6*-Cre mice (stock no. 011038) and PGK-Cre global deleter mice (stock no. 020811) were obtained from The Jackson Laboratory. Fibroblast-specific tamoxifen-inducible *Col1a2*-Cre-ER(T) mice have been well characterized (17, 37) and were provided in-house.

#### Breeding of cardiomyocyte-specific and fibroblast-specific KO mice.

The breeding strategy to obtain cardiomyocyte-specific IL-1α–KO mice (Supplemental Figure 1B) involved crossing homozygous *Il1a*^fl/fl^ mice with heterozygous *Myh6*-Cre mice to generate *Myh6*-Cre-*Il1a*^fl/fl^ mice. When these were backcrossed with *Il1a*^fl/fl^ mice, Cre-positive cardiomyocyte-specific IL-1α–KO mice (MIL1AKO mice) and Cre-negative floxed littermates (control) were produced.

For investigating IL-1R1 in cardiac fibroblasts, a tamoxifen-inducible fibroblast-specific IL-1R1–KO mouse line [*Col1a2*-CreER(T)-*Il1r1*^fl/fl^] was initially established on a C57BL/6 background by crossing *Col1a2*-CreER(T) mice with *Il1r1*^fl/fl^ mice (Supplemental Figure 3A). Global IL-1R1–KO mice were subsequently established by crossing female PGK-Cre mice with male *Il1r1*^fl/fl^ mice on a C57BL/6 background (Supplemental Figure 3B). Finally, a tamoxifen-inducible fibroblast-specific IL-1R1 hemizygous KO mouse line (FIL1R1KO) [*Col1a2*-CreER(T)-*Il1r1*^fl/–^] was established by crossing heterozygous *Col1a2*-Cre-ER(T) mice with *Il1r1*^fl/fl^ mice on a C57BL/6 background (Supplemental Figure 3C).

### Tamoxifen-inducible Cre activation

For the fibroblast-targeted studies, control (Cre-negative *Il1r1*^fl/–^) and FIL1R1KO mice were injected with tamoxifen dissolved in corn oil (100 mg/kg/d i.p. for 5 consecutive days) at 12 days of age to induce CreER(T) activity and facilitate Cre/loxP-mediated deletion in Cre-positive fibroblasts.

### Genotyping PCR

For routine genotyping, DNA was extracted from lysed ear notch/tissue samples using tail lysis buffer supplemented with proteinase K as described previously (20), and endpoint PCR was performed with specific primer pairs (Supplemental Table 3) before resolution by agarose gel electrophoresis (Figure 1B, Supplemental Figure 1A, and Supplemental Figure 4). Routine genotyping was subsequently outsourced to Transnetyx for automated analysis.

Endpoint PCR was also used to confirm cardiomyocyte-specific deletion of *Il1a* exon 4 in whole heart tissue and fibroblast-specific deletion of *Il1r1* exon 5 in cultured cardiac fibroblasts using specific primer sets (Supplemental Table 3).

### Experimental MI model

A permanent LAD coronary artery ligation model of experimental MI was performed on 5% isoflurane-anesthetized male mice (10–12 weeks of age), as we described previously (38). Following intubation, mice were ventilated at a tidal volume of 140 μl and a respiratory rate of 120/minute with 1.5% isoflurane and 100% oxygen. The LAD was ligated at the edge of the left atrium using 8-0 prolene suture, and occlusion was confirmed by observing the pallor of the anterior LV wall.

For cardiomyocyte-targeted mice, LAD ligation was performed on Cre-positive (MIL1AKO) and Cre-negative (control) littermates. For fibroblast-targeted mice, LAD ligation was performed on Cre-positive (FIL1R1KO) and Cre-negative (control) littermates that had been injected with tamoxifen at 12 days of age. Sham-operated animals (no tamoxifen treatment) underwent a similar surgical procedure without tying the ligature. In order to keep the number of animals used to a minimum, sham data were combined from the 2 different genotypes (Cre-positive and Cre-negative), which showed no differences in baseline phenotype.

### Measurement of cardiac function

Physiological measurements of cardiac function were obtained by Millar conductance P-V catheter analysis, as we have described in full previously (20, 39). The investigator performing the P-V measurements was blinded to the genotype of the animals.

### Histology

Hearts were extracted at various time points and fixed in 4% paraformaldehyde before paraffin wax embedding. Tissue sections (5 μm) were stained as we described previously with Miller’s elastic van Gieson (MVG) stain (40) or with antibodies for tenascin-C (Santa Cruz Biotechnology, sc-8694) (38). Composite tiled images were prepared from individual photomicrographs so that cross-sections through the whole heart could be observed.

### Real-time qRT-PCR

RNA was purified from cells or snap-frozen heart tissue, and cDNA synthesis was performed as described previously (20). Real-time qRT-PCR was undertaken using an ABI-7500 System with specific TaqMan primer/probe sets (Applied Biosystems) for detecting *Acta2* (Mm00725412_s1), *Col1a1* (Mm00801666_g1), *Col3a1* (Mm01254476_m1), *Gapdh* (Mm99999915_g1), *Il1a* (Mm99999060_m1), *Il1b* (Mm00434228_m1), *Il1r1* (Mm01226961), *Il6* (Mm00446190_m1), *Mmp3* (Mm00440295_m1), *Mmp9* (Mm00442991_m1), *Myh7* (Mm00600555_m1), *Nppa* (Mm01255747_g1), *Postn* (Mm01284919_m1), *Tnc* (Mm00495662_m1), and *Tnfa* (Mm00443258_m1). Data are expressed relative to *Gapdh* mRNA expression using the 2^–ΔCT^ method.

### Cardiac fibroblast culture

Cardiac fibroblasts were cultured from mouse hearts by a collagenase digestion method, as we described previously (20, 41). Experiments were performed in serum-free medium using early passage (P0 or P1) cells.

### Immunoblotting

Immunoblotting was performed as described previously (42) using Cell Signaling Technology antibodies for phospho-p38 (product 9211) and IκB-α expression (product 9242). β-Actin expression (loading control) was detected with anti-β actin antibody (Abcam, ab8226). Horseradish peroxidase–conjugated secondary antibodies and enhanced chemiluminescence detection reagent were from GE Healthcare. Blots were imaged using the G:BOX gel doc system (Syngene), and band intensities were quantified using GeneTools software (Syngene) (Figure 1B, Supplemental Figure 1A, and Supplemental Figure 4).

### Statistics

Statistical analyses were conducted using GraphPad Prism 7.01 Software. Unless stated otherwise, data are mean ± SEM. Box-and-whisker plots illustrate median, 25th/75th percentiles, and minimum/maximum. The *n* value represents the number of separate animals or the number of cell cultures from separate hearts (i.e., biological replicates). Data were analyzed by 2-tailed *t* test, 2-way ANOVA (with Bonferroni post hoc), or 1-way ANOVA (with Tukey post hoc), as appropriate. A *P* value of less than 0.05 was considered significant.

### Study approval

All animal procedures were carried out in accordance with the Animal Scientific Procedures Act (United Kingdom) 1986 under United Kingdom Home Office authorization, following review and approval by the University of Leeds Animal Welfare and Ethical Review Committee.

## Author contributions

SAB, KEH, MJD, and NAT designed the experiments. SAB, KEH, MJD, AM, NYY, FOGE, NEH, and MSJ conducted the experiments. SAB, KEH, MJD, and NAT analyzed the data. CPD and EP provided reagents or tools. NAT, MJD, EP, SF, JFXA, and KEP obtained funding for this work. NAT conceptualized the study and wrote the manuscript. All authors commented on and approved the submission of this work.

## Supplementary Material

Supplemental data

## Version 1 08/08/2019

In-Press Preview

## Version 2 09/05/2019

Electronic publication

## References

[1] Chen W, Frangogiannis NG (2013). Fibroblasts in post-infarction inflammation and cardiac repair. Biochim Biophys Acta.

[2] Turner NA (2016). Inflammatory and fibrotic responses of cardiac fibroblasts to myocardial damage associated molecular patterns (DAMPs). J Mol Cell Cardiol.

[3] Turner NA (2014). Effects of interleukin-1 on cardiac fibroblast function: relevance to post-myocardial infarction remodelling. Vascul Pharmacol.

[4] Frangogiannis NG (2015). Interleukin-1 in cardiac injury, repair, and remodeling: pathophysiologic and translational concepts. Discoveries (Craiova).

[5] Guillén I, Blanes M, Gómez-Lechón MJ, Castell JV (1995). Cytokine signaling during myocardial infarction: sequential appearance of IL-1 beta and IL-6. Am J Physiol.

[6] Buckley LF, Abbate A (2018). Interleukin-1 blockade in cardiovascular diseases: a clinical update. Eur Heart J.

[7] Huang S, Frangogiannis NG (2018). Anti-inflammatory therapies in myocardial infarction: failures, hopes and challenges. Br J Pharmacol.

[8] Libby P (2017). Interleukin-1 Beta as a Target for Atherosclerosis Therapy: Biological Basis of CANTOS and Beyond. J Am Coll Cardiol.

[9] Chen CJ, Kono H, Golenbock D, Reed G, Akira S, Rock KL (2007). Identification of a key pathway required for the sterile inflammatory response triggered by dying cells. Nat Med.

[10] Lugrin J (2015). Cutting edge: IL-1α is a crucial danger signal triggering acute myocardial inflammation during myocardial infarction. J Immunol.

[11] Mauro AG (2017). Reduction of Myocardial Ischemia-Reperfusion Injury by Inhibiting Interleukin-1 Alpha. J Cardiovasc Pharmacol.

[12] Martín-Sánchez F (2016). Inflammasome-dependent IL-1β release depends upon membrane permeabilisation. Cell Death Differ.

[13] Weber A, Wasiliew P, Kracht M (2010). Interleukin-1 (IL-1) pathway. Sci Signal.

[14] Bujak M (2008). Interleukin-1 receptor type I signaling critically regulates infarct healing and cardiac remodeling. Am J Pathol.

[15] Saxena A (2013). IL-1 induces proinflammatory leukocyte infiltration and regulates fibroblast phenotype in the infarcted myocardium. J Immunol.

[16] Abbate A (2011). Alterations in the interleukin-1/interleukin-1 receptor antagonist balance modulate cardiac remodeling following myocardial infarction in the mouse. PLoS ONE.

[17] Denton CP (2009). Inducible lineage-specific deletion of TbetaRII in fibroblasts defines a pivotal regulatory role during adult skin wound healing. J Invest Dermatol.

[18] Lal H (2014). Cardiac fibroblast glycogen synthase kinase-3β regulates ventricular remodeling and dysfunction in ischemic heart. Circulation.

[19] Ubil E (2014). Mesenchymal-endothelial transition contributes to cardiac neovascularization. Nature.

[20] Bageghni SA (2018). Cardiac fibroblast-specific p38α MAP kinase promotes cardiac hypertrophy via a putative paracrine interleukin-6 signaling mechanism. FASEB J.

[21] Feil S, Valtcheva N, Feil R. 2009. Inducible Cre mice. In: Kuhn R, Wurst W, eds. *Gene Knockout Protocols*. New York, New York, USA: Humana Press; 2009:343–363.

[22] Nishikawa K (2006). Left ventricular hypertrophy in mice with a cardiac-specific overexpression of interleukin-1. Am J Physiol Heart Circ Physiol.

[23] Pinto AR (2016). Revisiting Cardiac Cellular Composition. Circ Res.

[24] Toldo S (2013). Interleukin-1β blockade improves cardiac remodelling after myocardial infarction without interrupting the inflammasome in the mouse. Exp Physiol.

[25] Harouki N (2017). The IL-1β Antibody Gevokizumab Limits Cardiac Remodeling and Coronary Dysfunction in Rats With Heart Failure. JACC Basic Transl Sci.

[26] Christia P (2013). Systematic characterization of myocardial inflammation, repair, and remodeling in a mouse model of reperfused myocardial infarction. J Histochem Cytochem.

[27] Abbate A (2013). Effects of interleukin-1 blockade with anakinra on adverse cardiac remodeling and heart failure after acute myocardial infarction [from the Virginia Commonwealth University-Anakinra Remodeling Trial (2) (VCU-ART2) pilot study]. Am J Cardiol.

[28] Morton AC (2015). The effect of interleukin-1 receptor antagonist therapy on markers of inflammation in non-ST elevation acute coronary syndromes: the MRC-ILA Heart Study. Eur Heart J.

[29] Abbate A (2010). Interleukin-1 blockade with anakinra to prevent adverse cardiac remodeling after acute myocardial infarction (Virginia Commonwealth University Anakinra Remodeling Trial [VCU-ART] Pilot study). Am J Cardiol.

[30] Van Tassell BW (2018). IL-1 Blockade in Patients With Heart Failure With Preserved Ejection Fraction. Circ Heart Fail.

[31] Ridker PM (2017). Antiinflammatory Therapy with Canakinumab for Atherosclerotic Disease. N Engl J Med.

[32] Pugach EK, Richmond PA, Azofeifa JG, Dowell RD, Leinwand LA (2015). Prolonged Cre expression driven by the α-myosin heavy chain promoter can be cardiotoxic. J Mol Cell Cardiol.

[33] Fan D (2016). A Disintegrin and Metalloprotease-17 Regulates Pressure Overload-Induced Myocardial Hypertrophy and Dysfunction Through Proteolytic Processing of Integrin β1. Hypertension.

[34] Skarnes WC (2011). A conditional knockout resource for the genome-wide study of mouse gene function. Nature.

[35] Gertsenstein M (2010). Efficient generation of germ line transmitting chimeras from C57BL/6N ES cells by aggregation with outbred host embryos. PLoS ONE.

[36] Abdulaal WH (2016). Characterization of a conditional interleukin-1 receptor 1 mouse mutant using the Cre/LoxP system. Eur J Immunol.

[37] Zheng B, Zhang Z, Black CM, de Crombrugghe B, Denton CP (2002). Ligand-dependent genetic recombination in fibroblasts: a potentially powerful technique for investigating gene function in fibrosis. Am J Pathol.

[38] Maqbool A (2016). Tenascin C upregulates interleukin-6 expression in human cardiac myofibroblasts via toll-like receptor 4. World J Cardiol.

[39] Frentzou GA, Drinkhill MJ, Turner NA, Ball SG, Ainscough JF (2015). A state of reversible compensated ventricular dysfunction precedes pathological remodelling in response to cardiomyocyte-specific activity of angiotensin II type-1 receptor in mice. Dis Model Mech.

[40] Yuldasheva NY (2014). Haploinsufficiency of the insulin-like growth factor-1 receptor enhances endothelial repair and favorably modifies angiogenic progenitor cell phenotype. Arterioscler Thromb Vasc Biol.

[41] Mylonas KJ (2017). 11β-HSD1 suppresses cardiac fibroblast CXCL2, CXCL5 and neutrophil recruitment to the heart post MI. J Endocrinol.

[42] Turner NA, Ball SG, Balmforth AJ (2001). The mechanism of angiotensin II-induced extracellular signal-regulated kinase-1/2 activation is independent of angiotensin AT(1A) receptor internalisation. Cell Signal.

