# Supplementary material for: Fibroblast-specific deletion of IL-1 receptor-1 reduces adverse cardiac remodeling following myocardial infarction
Source: JCI Insight. 2019 Sep 5;4(17):e125074. doi: 10.1172/jci.insight.125074 (PMC6777910; doi:10.1172/jci.insight.125074)
Supplement: Supplemental data [file jciinsight-4-125074-s128.pdf]

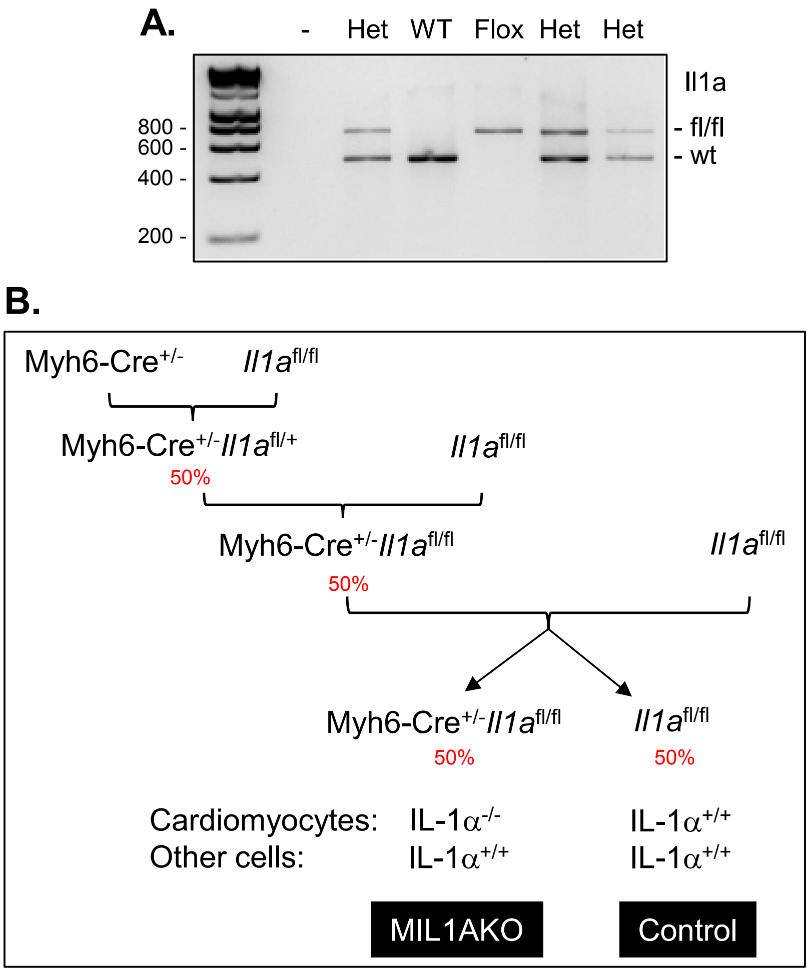

Supplemental Figure 1. Genotyping and breeding strategy for generating the cardiomyocyte-specific IL-1α knockout mouse line (MIL1AKO). (A) A floxed mouse line (*Il1a*$^{fl/fl}$) was generated in which exon 4 of the *Il1a* gene was flanked by loxP sites. Genotyping PCR showing amplification of wild-type and *Il1a*$^{fl/fl}$ alleles in wild-type (WT; +/+; 485 bp), heterozygous (Het; +/fl) and floxed (fl/fl; 692 bp) mice. Size markers (bp) are to the left. See Supplemental Table 3 for primer details. **(B)** Breeding strategy for generating *Myh6*-Cre-*Il1a*$^{fl/fl}$ (MIL1AKO) line. Floxed *Il1a* mice (*Il1a*$^{fl/fl}$) were bred with heterozygous mice expressing Cre recombinase under control of the cardiomyocyte-specific *Myh6* promoter (*Myh6*-Cre$^{+/-}$) to produce Cre-positive cardiac myocyte-specific IL-1α KO (MIL1AKO) mice and control Cre-negative floxed littermates. Percentages indicate predicted proportion of correct genotype.

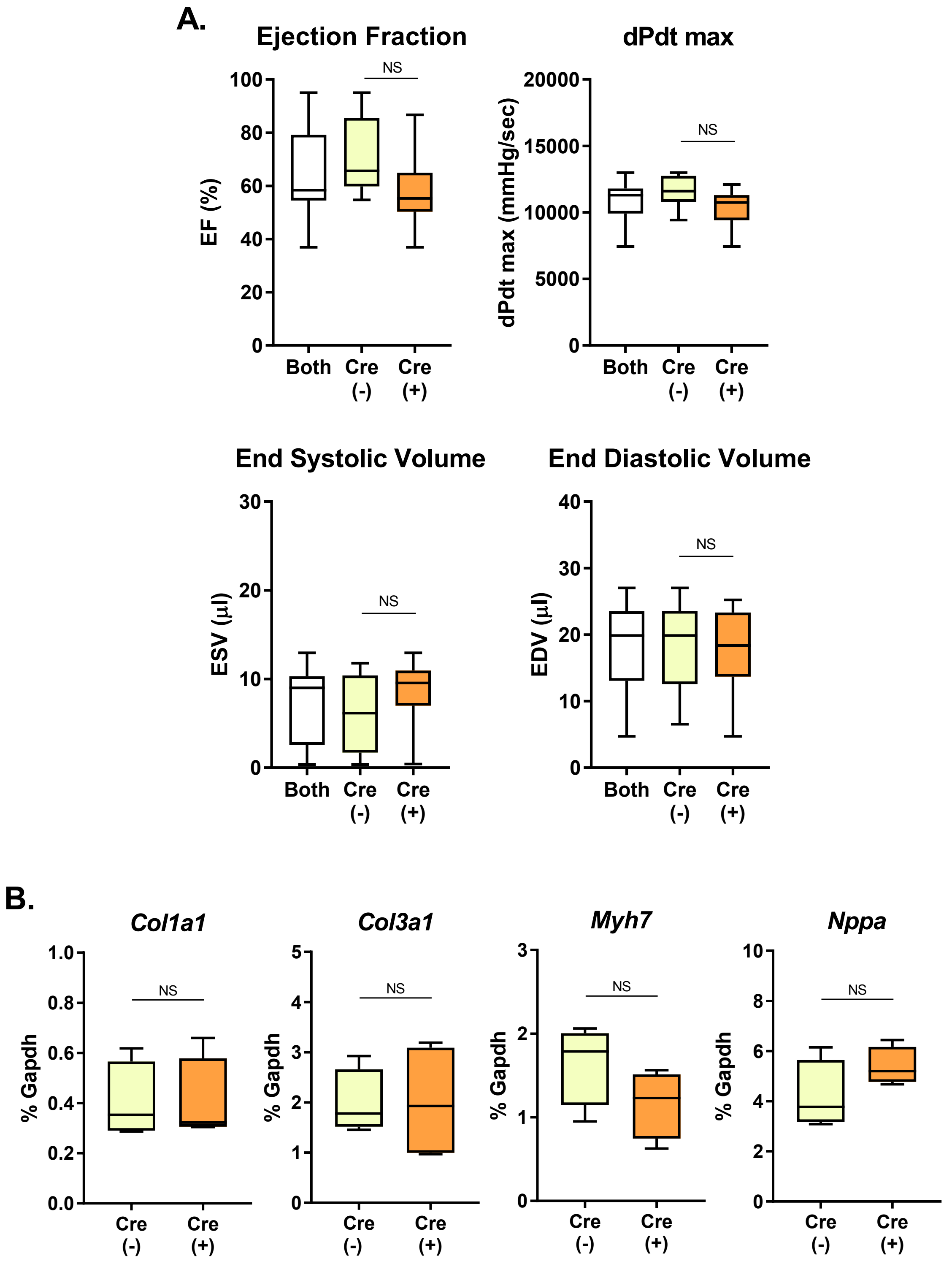

Supplemental Figure 2. Comparison of baseline cardiac characteristics and gene expression in myocyte-specific Cre lines. (A) Cardiac parameters assessed by Millar pressure-volume conductance catheter in sham operated animals. Cre(-) = Cre-negative *Il1a*$^{fl/fl}$ (n=9); Cre(+) = *Myh6*-Cre-positive *Il1a*$^{fl/fl}$ (n=10); Both = combined (n=19), as shown in Figure 1E. Box plots illustrate median, 25th/75th percentiles and minimum/maximum. NS = not significantly different between Cre(-) and Cre(+) (2-tailed unpaired t-test). **(B)** qRT-PCR data showing relative mRNA levels of remodeling genes collagen Iα1 (*Col1a1*), collagen IIIα1 (*Col3a1*), β-myosin heavy chain (*Myh7*) and atrial natriuretic factor (*Nppa*) in hearts from sham operated animals. Cre(-) = Cre-negative *Il1a*$^{fl/fl}$ (n=4); Cre(+) = *Myh6*-Cre-positive *Il1a*$^{fl/fl}$ (n=4). NS = not significantly different (2-tailed unpaired t-test).

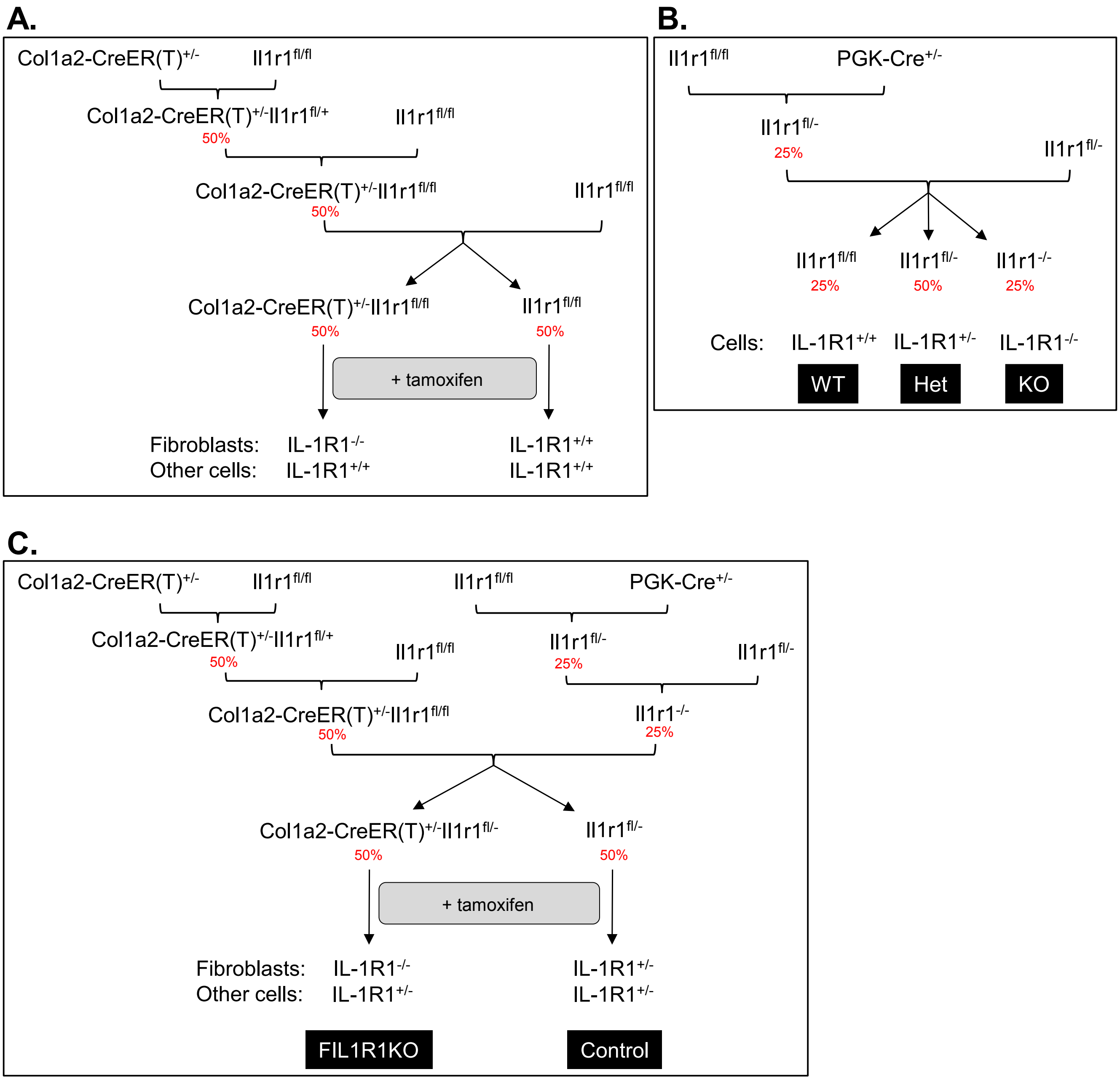

Supplemental Figure 3. Breeding strategies for generating fibroblast-targeted mouse lines. (A) Generation of *Col1a2*-CreER(T)-*Il1r1*$^{fl/fl}$ line. Floxed *Il1r1* mice (*Il1r1*$^{fl/fl}$) were bred with heterozygous mice expressing tamoxifen-inducible Cre recombinase under control of the fibroblast-specific *Col1a2* promoter (*Col1a2*-CreER(T)$^{+/-}$). After tamoxifen treatment, this resulted in Cre-positive fibroblast-specific IL-1R1 KO mice and control Cre-negative *Il1r1* floxed littermates. **(B)** Generation of IL-1R1$^{+/-}$ and IL-1R1$^{-/-}$ lines. Male floxed *Il1r1* mice were bred with female PGK-Cre global deleter mice to produce *Il1r1*$^{fl/-}$ mice. These were back crossed with other *Il1r1*$^{fl/-}$ mice to generate a combination of *Il1r1*$^{+/+}$ (WT), *Il1r1*$^{fl/-}$ (Het) and *Il1r1*$^{-/-}$ (KO). **(C)** Generation of FIL1R1KO line. *Col1a2*-CreER(T)$^{+/-}$*Il1r1*$^{fl/fl}$ mice were crossed with *Il1r1*$^{-/-}$ mice to generate the Cre-positive *Col1a2*-CreER(T)-*Il1r1*$^{fl/-}$ line. After tamoxifen treatment, this resulted in a fibroblast-specific IL1R1 KO (FIL1R1KO). Control animals were Cre-negative hemizygous floxed (*Il1r1*$^{fl/-}$) littermates. Percentages indicate predicted proportion of correct genotype.

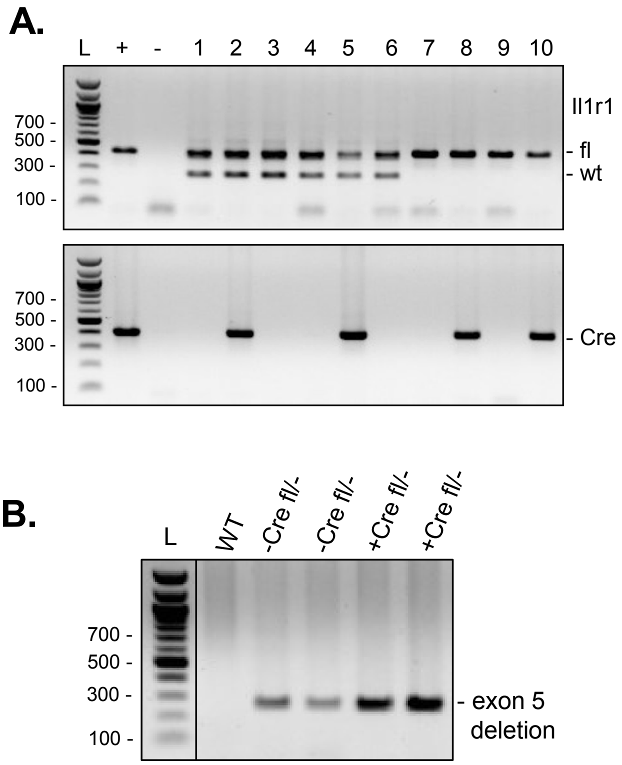

Supplemental Figure 4. Genetic characterization of tamoxifen-inducible fibroblast-specific IL-1R1 knockout mice. (A) Genotyping of *Il1r1*$^{fl/fl}$ lines. L = 100 bp ladder; +/- = positive/negative control. Upper panel: *Il1r1* fl primers; floxed (fl; 432 bp), wild-type (wt; 267 bp). Lower panel: Cre primers (408 bp). **(B)** Genotyping for exon 5 deletion (280 bp) in *Il1r1*$^{fl/-}$ lines after tamoxifen injection. Vertical dividing line indicates that the samples were run on the same gel but were non-contiguous. See Supplemental Table 3 for primer details.

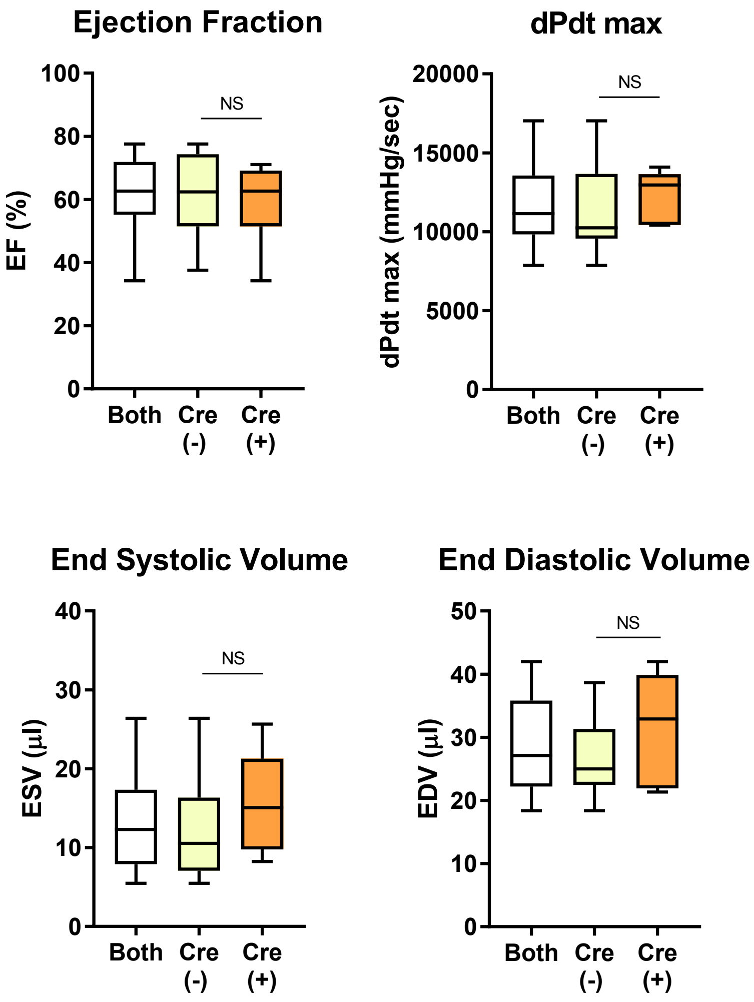

Supplemental Figure 5. Comparison of baseline cardiac characteristics of Cre-negative and Cre-positive IL-1R1 floxed mice. Cardiac parameters were assessed by Millar pressure-volume conductance catheter in sham operated animals. Cre(-) = Cre-negative *Il1r1*$^{fl/-}$ (n=12); Cre(+) = *Col1a2*-Cre-positive *Il1r1*$^{fl/-}$ (n=6); Both = combined (n=18), as shown in Figure 5B. Neither group was injected with tamoxifen. Box plots illustrate median, 25th/75th percentiles and minimum/maximum. NS = not significantly different between Cre(-) and Cre(+) (2-tailed unpaired t-test).

Parameter	Sham (n=19)	MI Ctrl (n=19)	MI MIL1AKO (n=17)
**Ejection fraction (%)**	64.57 ± 3.65	40.76 ± 3.31***	42.86 ± 9.85***
**End systolic volume (µl)**	7.35 ± 1.02	18.18 ± 1.49***	16.13 ± 1.64***
**End diastolic volume (µl)**	17.82 ± 1.50	29.31 ± 1.26***	26.78 ± 1.96***
**Cardiac output (µl/min)**	6195 ± 402	6352 ± 491	5893 ± 450
**Stroke volume (µl)**	11.52 ± 0.78	11.58 ± 0.89	11.32 ± 0.89
**Heart rate (bpm)**	541.6 ± 9.2	555.6 ± 14.8	524.6 ± 12.7
**End systolic pressure (mmHg)**	94.92 ± 2.59	104.4 ± 1.76**	99.41 ± 1.85
**End diastolic pressure (mmHg)**	4.42 ± 0.94	8.09 ± 1.22	6.55 ± 1.19
**$dPdt_{max}$ (mmHg/sec)**	10911 ± 340	10200 ± 503	8952 ± 272**
**$dPdt_{min}$ (mmHg/sec)**	-10474 ± 391	-9768 ± 586	-8607 ± 313*
**Arterial blood pressure (mmHg)**	70.7 ± 1.6	74.7 ± 1.9	73.3 ± 1.8

Supplemental Table 1. Effect of cardiomyocyte-specific IL-1α deletion on cardiac function 4 weeks after myocardial infarction. Cardiac parameters were assessed by Millar pressure-volume conductance catheter. Sham = mixed genotypes (n=19); MI Ctrl = Cre-negative *Il1a*$^{fl/fl}$ after MI (n=19); MI MIL1AKO = *Myh6*-Cre-positive *Il1a*$^{fl/fl}$ after MI (n=17). Data are mean values ± SEM. ***P<0.001, **P<0.01, *P<0.05 versus sham control (1-way ANOVA with Tukey post hoc). All other comparisons not significant. Note that ejection fraction, end systolic volume, end diastolic volume and $dPdt_{max}$ data are also depicted in Figure 1E.

Parameter	Sham (n=18)	MI Ctrl (n=11)	MI FIL1R1KO (n=11)
Ejection fraction (%)	61.24 ± 2.93	47.84 ± 3.47*	56.03 ± 3.68
End systolic volume (µl)	13.36 ± 1.50	18.83 ± 2.03	14.53 ± 2.46
End diastolic volume (µl)	28.54 ± 1.76	31.68 ± 2.49	27.15 ± 2.77
Cardiac output (µl/min)	10195 ± 576	8562 ± 1052	7742 ± 553
Stroke volume (µl)	17.68 ± 0.97	15.41 ± 1.59	14.46 ± 0.98
Heart rate (bpm)	579.9 ± 16.5	550.3 ± 18.4	536.3 ± 17.6
End systolic pressure (mmHg)	104.40 ± 2.92	91.45 ± 3.46*	93.36 ± 2.38*
End diastolic pressure (mmHg)	9.88 ± 1.25	9.68 ± 2.17	10.81 ± 1.17
$dPdt_{max}$ (mmHg/sec)	11805 ± 574	9350 ± 747*	9828 ± 391
$dPdt_{min}$ (mmHg/sec)	-11091 ± 561	-9386 ± 944	-9251 ± 563
Arterial blood pressure (mmHg)	71.6 ± 2.4	67.6 ± 2.1	67.4 ± 1.5

Supplemental Table 2. Effect of fibroblast-specific IL-1R1 deletion on cardiac function 4 weeks after myocardial infarction. Sham = mixed genotypes, no tamoxifen treatment (n=18); MI Ctrl = tamoxifen-treated Cre-negative *Il1r1*$^{fl/-}$ after MI (n=11); MI FIL1R1KO = tamoxifen-treated *Col1a2*-Cre-positive *Il1r1*$^{fl/-}$ after MI (n=11). Data are mean values ± SEM. *P<0.05 versus sham control (1-way ANOVA with Tukey post hoc). All other comparisons not significant. Note that ejection fraction, end systolic volume, end diastolic volume and $dPdt_{max}$ data are also depicted in Figure 5B.

Primer target	Primer sequence	Product size (bp)
Cre (forward)<br>Cre (reverse)	5’ GCATTACCGGTCGATGCAACGAGTGATGAG 3’<br>5’ GAGTGAACGAACCTGGTCGAAATCAGTGCG 3’	408 (Cre)
Il1a fl (forward)<br>*Il1a* fl (reverse)	5’ TTGTCCTCCCTAGCCAGTTG 3’<br>5’ ATTTGGCCCTCTGTTTTGCC 3’	692 (floxed)<br>485 (wild type)
Il1a del (forward)<br>*Il1a* del (reverse)	5’ TTGTCCTCCCTAGCCAGTTG 3’<br>5’ TCCAGTGCTGAAAAGTGTGC 3’	456 (deletion)
Il1r1 fl (forward)<br>*Il1r1* fl (reverse)	5’ CTAGTCTGGTGGAACTTACATGC 3’<br>5’ AACTGAAAGCTCAGTTGTATACAGC 3’	432 (floxed)<br>267 (wild type)
Il1r1 del (forward)<br>*Il1r1* del (reverse)	5’ CTAGTCTGGTGGAACTTACATGC 3’<br>5’ GATAAAGCAGAGCTGGAGACAGG 3’	280 (deletion)

Supplemental Table 3. Genotyping of transgenic mice. Properties of PCR primers used for genotyping and predicted product sizes. bp=base pairs.

Areas shown in final figure

Full unedited gel for Figure 1B (upper panel):

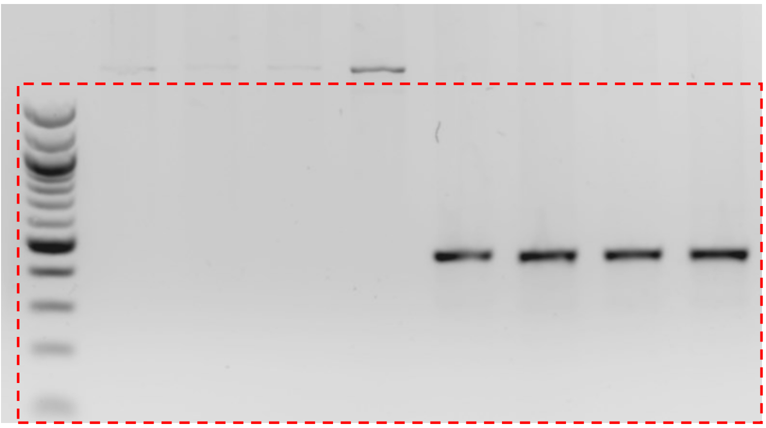

Full unedited gel for Figure 1B (lower panel):

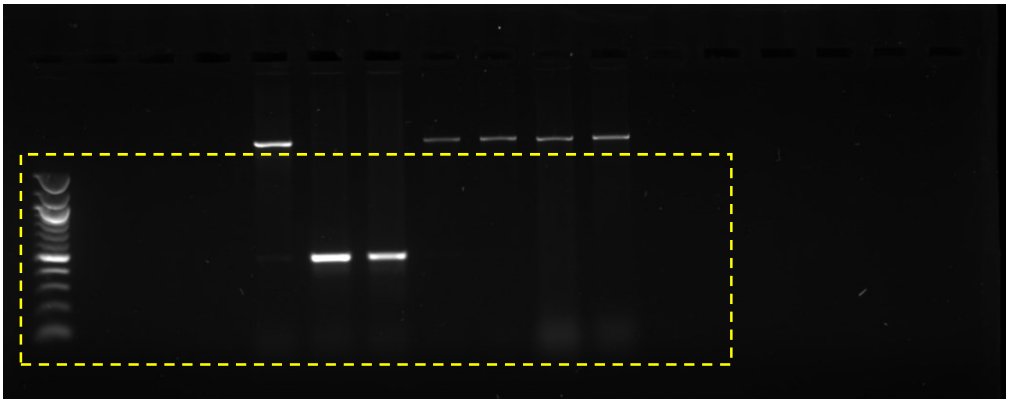

Black/white inverted for consistency

Full unedited gels for Figure 3B:

Areas shown in final figure

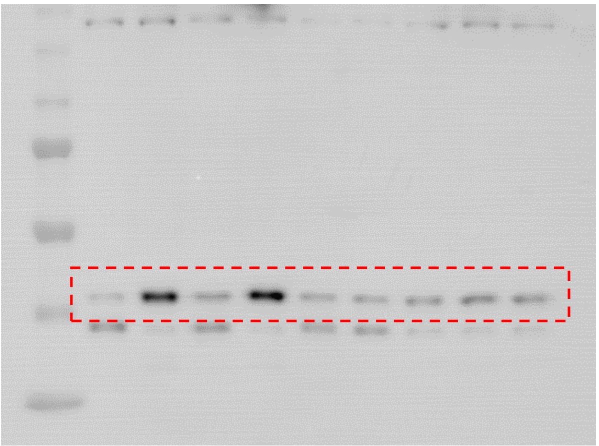

Lower bands are from previous probing with different primary antibody (IkB expression - image below)

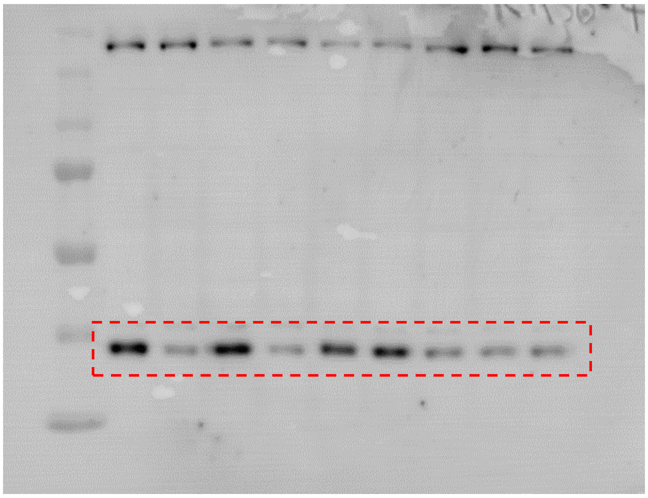

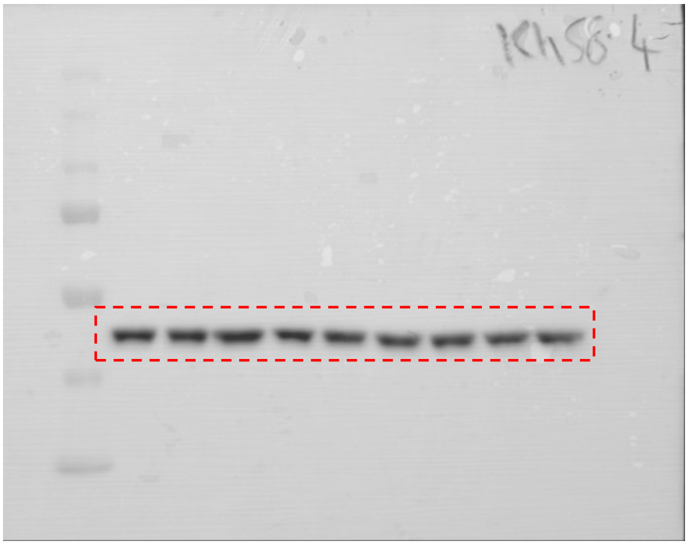

Full unedited gels for Figure 4C:

Areas shown in final figure

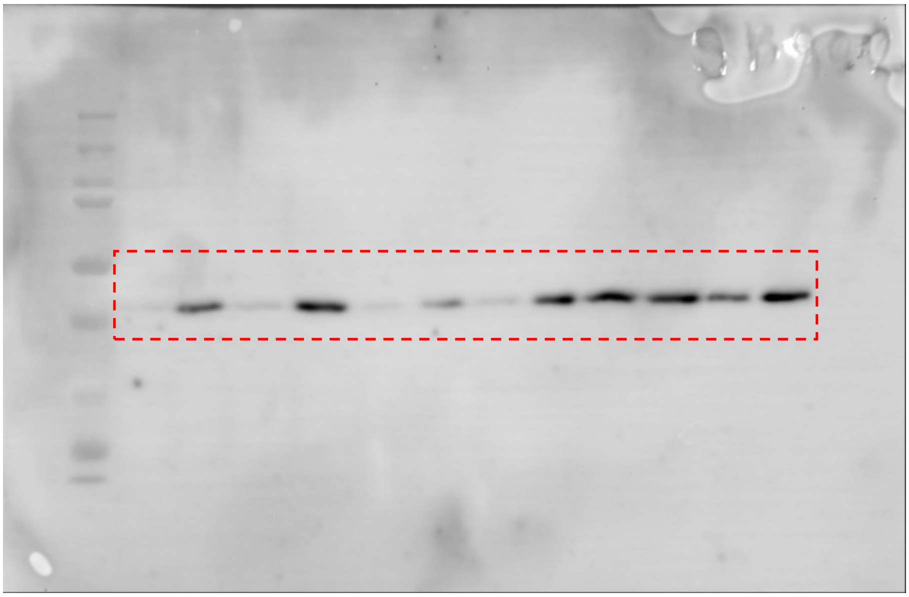

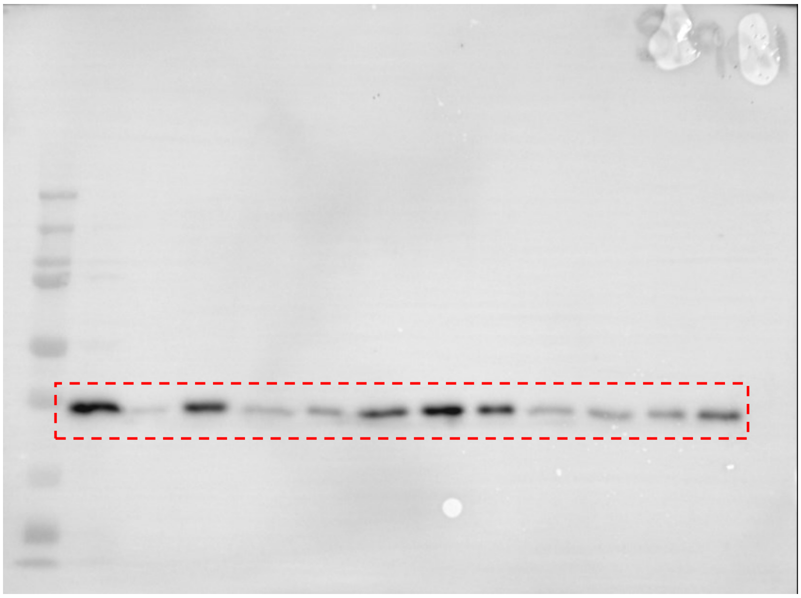

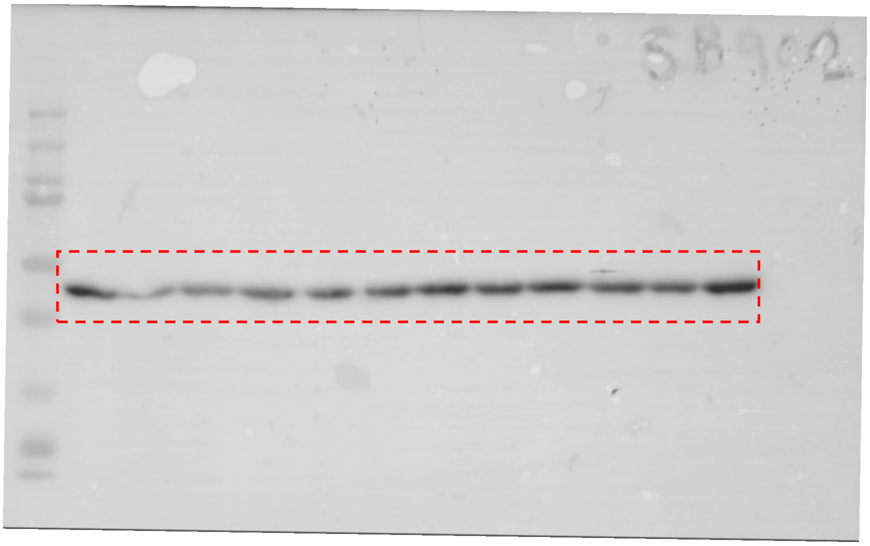

Areas shown in final figure

Full unedited gel for Supplemental Figure 1A:

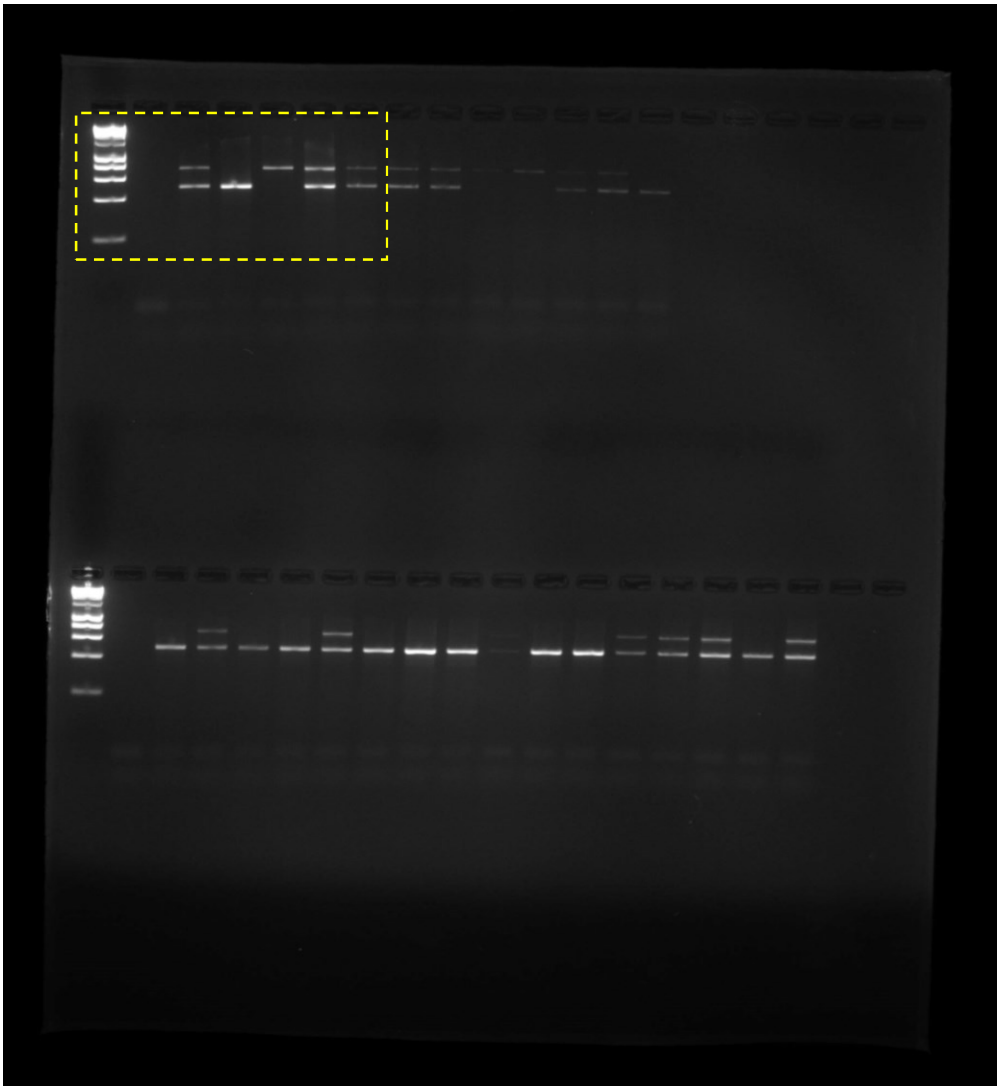

Black/white inverted for consistency

Areas shown in final figure

Full unedited gels for Supplemental Figure 4A:

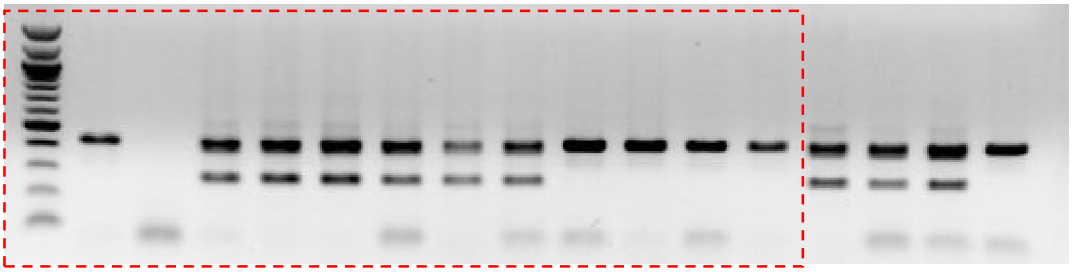

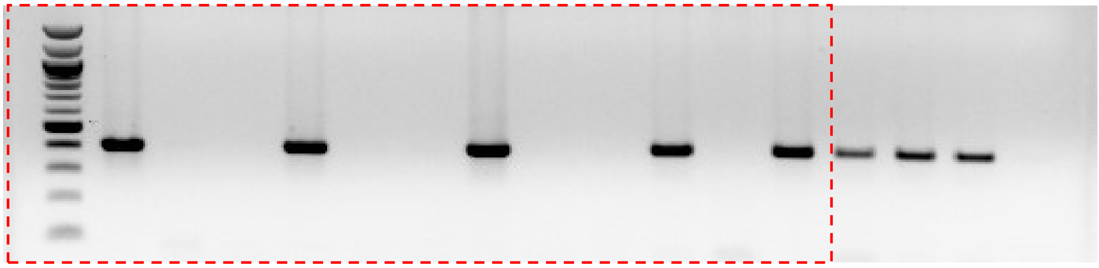

Full unedited gel for Supplemental Figure 4B:

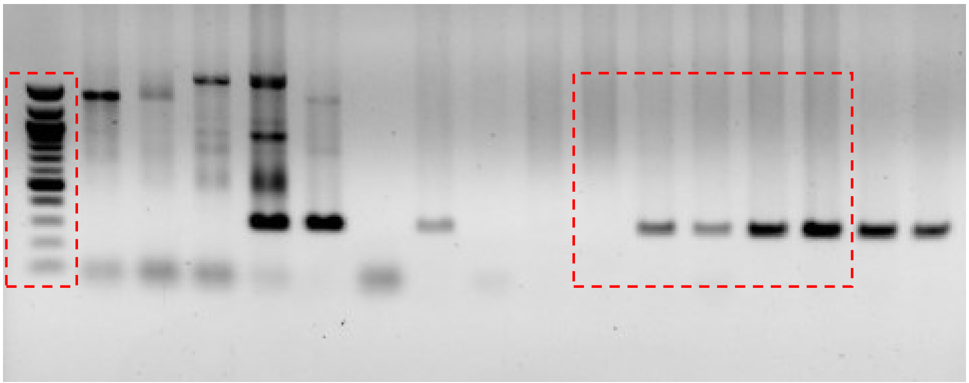

Size ladder moved adjacent to samples for convenience
